# Prominent astrocytic GLAST pathology occurs in newborn human and piglet hypoxic–ischemic encephalopathy: modeling relationships among laminar neuropathology, seizures, and therapeutic hypothermia

**DOI:** 10.3389/fncel.2026.1758411

**Published:** 2026-02-24

**Authors:** Dongseok Park, Caitlin E. O’Brien, Jennifer K. Lee, Lee J. Martin

**Affiliations:** 1 Department of Pathology, Johns Hopkins University School of Medicine, Baltimore, MD, United States; 2 Department of Anesthesiology and Critical Care Medicine, Johns Hopkins University School of Medicine, Baltimore, MD, United States; 3 Department of Neuroscience, Johns Hopkins University School of Medicine, Baltimore, MD, United States; 4 The Pathobiology Graduate Training Program, Johns Hopkins University School of Medicine, Baltimore, MD, United States

**Keywords:** cEEG, human HI, neonatal piglet HIE, seizure, therapeutic hypothermia

## Abstract

Neonatal hypoxic–ischemic encephalopathy (HIE) is frequently complicated by seizures that persist despite therapeutic hypothermia (HT), suggesting injury mechanisms insensitive to HT. Here, we tested the hypothesis that astrocytic glutamate–aspartate transporter (GLAST) abnormalities in the neocortex contribute to cortical hyperexcitability and seizure burden after HIE, and that HT mitigates this astrocyte-mediated mechanism. We examined the vulnerability of GLAST in the neocortex of human neonatal hypoxic–ischemic encephalopathy (HIE) and in a piglet model of hypoxia-ischemia (HI). We determined how GLAST immunoreactivity localization associates with HT outcome in clinical and experimental settings. Brain sections from postmortem human autopsy cases of term neonatal HIE and piglets (2–3 days old, *n* = 5–6/group) were used to localize GLAST and glial fibrillary acidic proteins (GFAP) across cortical layer I-III in the somatosensory cortex. Piglets received continuous 4-channel epidural EEG recording under normothermia (NT) or mild HT (38 °C to 34 °C with rewarming; 29 h), with hypoxic-asphyxic cardiac arrest and resuscitation, or a sham procedure. Piglet survival was assessed over 7 days. Neuropathology was identified by the number of damaged neurons and GLAST puncta metrics. EEG-seizure metrics, including ictal event frequency, duration, spike–wave events, and power spectral density (PSD), were quantified using a custom seizure classification pipeline. GLAST localization in human HIE cortex was significantly abnormal compared to non-HIE control cases, characterized by perisomatic aggregation and reduced neuropil density. HI in piglets reproduced these GLAST abnormalities, including apparent aggregation, that correlated with seizure burden and neuronal pathology. HT attenuated the GLAST pathology in HI piglets at perisomatic locations to the level of sham, particularly in layers II-III, delayed seizure onset by ~24 h, and significantly reduced ictal event frequency (to lower than 5 events per 4 h) and duration (to less than 20 s per event). These findings identify prominent GLAST pathology in newborn humans and piglet HIE. HT partially restores astrocytic GLAST localization that is temporally associated with reduced seizure burden in piglets. We conclude that astrocytic glutamate transport abnormalities contribute to cortical hyperexcitability and seizures in neonatal HIE.

## Graphical abstract

**Figure:**
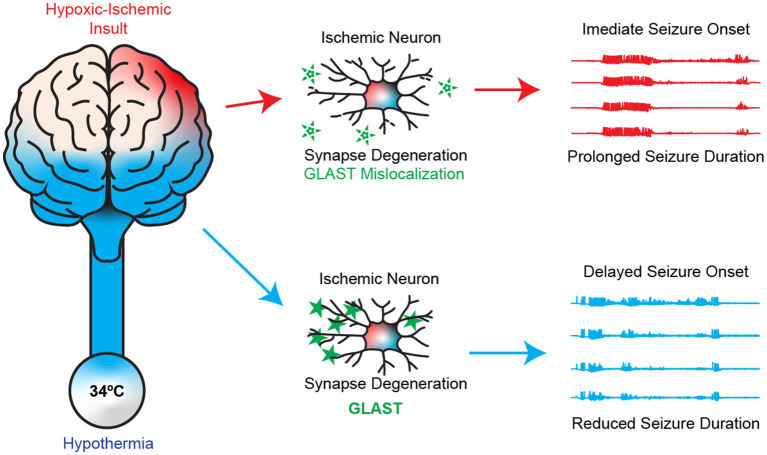
Autopsy brains of newborn patients with hypoxic–ischemic encephalopathy (HIE) show abnormal astrocytic GLAST localization, characterized by perisomatic aggregation and disrupted cortical organization. Experimentally, neonatal piglet hypoxia-ischemia recapitulates a cortical GLAST pathology that correlates with seizure burden and cortical neuron injury. Therapeutic hypothermia restores GLAST distribution, delays seizure onset, reduces seizure duration, and mitigates ischemic neurodegeneration in piglets. Astrocytic glutamate transport could be a mechanism for restoring normal cortical excitability in neonatal HIE with therapeutic hypothermia.

## Highlights

- Therapeutic hypothermia delays seizure onset following hypoxic–ischemic insult in piglets.
- Seizure burden correlates with ischemic neuronal loss in layer III of the somatosensory cortex.
- Power spectral density quantitatively distinguishes focal versus generalized seizure in piglets.
- Piglet HI reproduces human HIE GLAST pathology, linking astrocytic remodeling to seizures.
- Hypothermia restores GLAST localization, revealing astrocytic mechanisms of neuroprotection.

## Introduction

Neonatal hypoxic–ischemic encephalopathy (HIE) is a major cause of newborn death and long-term neurological disability. The current standard-of-care for moderate to severe neonatal HIE in some countries is therapeutic hypothermia (HT) because it improves survival and some neurological outcomes (Yap et al., 2009; Shankaran et al., 2005; Shankaran et al., 2012). Nevertheless, seizures remain a hallmark of HIE and can occur commonly during or after rewarming. They are frequently subclinical, often detectable only with continuous EEG (cEEG) (Glass et al., 2016; Shetty, 2015; Panayiotopoulos, 2005; Samanta, 2021; Tao and Mathur, 2010; Chalak et al., 2021). Neonatal seizure burden strongly correlates with injury severity (Schmid et al., 1999; Miller et al., 2002) and adverse neurodevelopmental outcomes; but whether seizures exacerbate hypoxic–ischemic (HI) brain injury or simply represent epiphenomena remains debated. Seizures might amplify excitotoxicity, oxidative stress, and metabolic failure to worsen neuronal loss, thus adding to the initial HI insult (Chalak et al., 2021; Glass et al., 2014) and contributing to poor outcomes (Miller et al., 2002; Glass et al., 2009). Alternatively, seizures could reflect the underlying insult with no additional injury (Kwon et al., 2011). This uncertainty needs resolution because (1) there is a suspected association between neonatal seizures and later neurodevelopmental impairments, and (2) commonly used anticonvulsants have limited efficacy (Zhou et al., 2021) and may themselves be a potential neurotoxicity (Bittigau et al., 2002; Forcelli et al., 2012; Noguchi et al., 2021) in infants.

A better understanding of the evolution of neonatal seizures after acquired brain injury, their neurobiological mechanisms, and how they relate to therapeutic HT and neuropathology is difficult to achieve in patients. Relevant experimental animal models could help, provided they share some key commonalities with term human HIE. However, many existing animal studies have not met these needs. Rodent models of neonatal HIE have been instrumental in defining molecular pathways of excitotoxic injury and neuroprotection (Li et al., 2025; Moreira et al., 2022; Cui et al., 2024; Pregnolato et al., 2022) but their utility in resolving laminar-specific astrocyte pathology and seizure evolution under clinically relevant injury and treatment conditions remains limited because of anatomical and physiological dissimilarities and insult modeling shortcomings. The term neonatal piglet has an anatomy and physiology that enable successful translation as a model of term human HIE (Koehler et al., 2018), but does not carry the profound cost and ethical heaviness of using infant non-human primates (Martin and Cork, 2014). Unlike rodents, piglets have a gyrencephalic cortex with adequately matured cortical lamination, discrete structural and functional localizations, human-like white to gray matter composition (Primiani et al., 2023), and developmental timing of neuronal and astrocytic maturation that more closely aligns with term infants, enabling region and layer-specific interrogation of injury mechanisms (Zhou et al., 2025; Allison et al., 2025; Landucci et al., 2022; She et al., 2023). Furthermore, the injury component has precise control and monitoring of cerebral perfusion, blood gases, and metabolic status under neonatal intensive care-like conditions, including mechanical ventilation, pharmacologic support, and therapeutic HT protocols like those used clinically (Allison et al., 2025; Kyng et al., 2015). Critically, the piglet model permits prolonged multichannel cEEG acquisition and synchronized neuropathological assessment, enabling direct temporal linkage between seizure evolution, cellular pathology, and treatment response capabilities that are limited or not feasible in rodent models. Together, these features position the neonatal piglets as a translational platform to better understand seizure mechanisms and therapeutic modulation in neonatal HIE. To address this gap between human clinical observations and limitations of rodent models, we used a piglet model to ascertain neuropathological similarities to human neonatal HIE and specifically identify links between laminar-specific astrocyte abnormalities and seizure evolution using continuous EEG, and examined whether HT can influence astrocytic and EEG perturbations after HI.

We hypothesize that HT mitigates astrocytic GLAST disruption, which correlates with stabilized cortical excitability and reduced neuronal injury. GLAST is a predominant astrocytic glutamate transporter during early development and functions in maintaining extracellular glutamate homeostasis (Furuta et al., 1997). Its dysregulation may contribute to excitotoxicity and network hyperexcitability underlying seizure generation in neonatal HIE. Our asphyxic cardiac arrest model using 2–3-day old piglets has controlled HI insult, precise temperature management for HT, continuous EEG monitoring, and quantitative postmortem neuropathological assessment (Primiani et al., 2023). The objective of this study was to (1) examine human HIE for GLAST abnormalities; (2) determine if similar pathology is present in our piglet model; and (3) investigate the relationships among GLAST pathology, seizure evolution, and HT. We found that HT mitigates astrocytic GLAST disruption, stabilizes cortical excitability, and reduces neuronal injury following neonatal HI. While these observation point toward a better cellular and molecular understanding of HIE and HT mechanisms of protection, the broader more immediate clinical importance of this work could be reflected by clinical proton magnetic resonance spectroscopy (1H-MRS) studies demonstrating elevated glutamate–glutamine levels in neonates with HIE that correlate positively with seizure severity (Pu et al., 2008) and the incorporation of metabolic imaging, including 1H-MRS, into postnatal assessment of seizure risk.

## Methods

### Human autopsy brain samples

Postmortem human brain samples were obtained from the Johns Hopkins University School of Medicine Brain Resource Center (Division of Neuropathology, Department of Pathology), the National Institute of Child Health and Development (NICHD) Brain and Tissue Bank for Developmental Disorders at the University of Maryland, Baltimore, MD, and the Children’s National Hospital, Washington, DC. All autopsies had approved consent, and tissues were de-identified. The protocols for using human autopsy tissue were reviewed and approved by the JHMI-IRB (N0. 02–09024-04e, approved 27 September 2002) and the Children’s National Hospital IRB (IRB#15350, approved 22 December 2020, and #11850, approved 17 January 2019). The human postmortem cases used are summarized in Table 1. The distant archival HIE cases predated HT as the standard of care, so these cases were normothermic. More recent HIE cases received HT protocols prior to death or missed the therapeutic opportunity for HT. Infantile human non-HIE cases of acute deaths due to non-neurological causes, such as accidental death, pneumonia, or drug intoxication, were used as comparators.

**Table 1:** Human infant autopsy cases used.

Case identifier	Age at birth (weeks)	Clinical setting	Therapeutic hypothermia	Age (at death)	Postmortem delay (h)
A15-7	36 (37)	Fetal deceleration and emergency C-section. Resuscitation involving chest compressions, cord blood pH6.9, and clinical seizures	Yes	7 days	24
A16-13	41.6 (42.9)	Shoulder dystocia; prolonged ruptured membranes	Yes	9 days	12
A16-30	34 (34.3)	Car collision, placental abruption, emergency C-section, DIC in newborn, neonatal respiratory failure	Yes	2 days	144
A17-1	39.3 (39.9)	Rupture of membranes, C-section, required chest compressions, cord blood pH 6.9	Yes	4 days	12
A17-3	38 (38.4)	Uncontrolled insulin-dependent diabetes (mother), emergency C-section, perinatal asphyxia, chest compressions	Yes	3 days	24
A17-14	24 (48.6)	Chronic lung disease with secondary pulmonary hypertension, hypoxemic respiratory failure, worsening hypotension	0 (missed therapeutic time window)	172 days	25
A18-2	35.3 (35.9)	Diamniotic dichorionic twins, premature rupture of membranes, emergency C-section, traumatic delivery (cephalohematoma, focal subdural hemorrhage, subarachnoid hemorrhages), chest compressions	Yes	4 days	16
A18-3	39.9 (40.4)	Secondary apnea, respiratory failure, multiorgan failure, possible septic shock	Yes	4 days	24
A18-17	34.3 (36.4)	HIV^+^ mother via emergency C section for fetal deceleration for systole, chest compressions	0 (missed therapeutic time window)	15 days	48
1047	Unavailable	Non-HIE control, accidental death	NA	7 years	24
993	Unavailable	Non-HIE control, accidental death.	NA	1 year	12
783	Unavailable	Non-HIE control, accidental death	NA	8 years	19
875	Unavailable	Non-HIE control, accidental death	NA	3 years	10
1176	Unavailable	Non-HIE control, accidental death	NA	20 years	6

### Animals

All experiments adhered to ARRIVE guidelines and were approved by the Institutional Animal Use and Care Committee of Johns Hopkins University (Protocol number SW23M119, approved June 6, 2023). Neonatal Yorkshire piglets (2–3 days old, 1–2 kg, males) were randomized to treatment groups using a randomization table to ensure unbiased assignment across experimental days and used for EEG recording at the somatosensory cortices and neuropathological assessments. The piglet treatment groups for the epidural EEG analysis in this study were: sham-normothermia (SH-NT, *n* = 4), sham-hypothermia (SH-HT, *n* = 6), HI-normothermia (HI-NT, *n* = 4), and HI-hypothermia (HI-HT, *n* = 4). All the piglets that had EEGs acquired were also used for neuropathology. Additional piglets used for neuropathology were in the SH-HT (*n* = 4), HI-NT (*n* = 6), and the HI-HT (*n* = 3 groups).

### Piglet global hypoxic-ischemia, mild HT, and cEEG acquisition

Piglet HI protocols and electrode placement are described in our previous publication (Primiani et al., 2023). Piglets were anesthetized with isoflurane and nitrous oxide in oxygen. Fentanyl was given as a bolus (20 ug/kg, iv), then as a continuous slow infusion (20 ug/kg/hr), with additional boluses as needed. Epidural bipolar 4-lead cEEG electrode arrays (Stellar Telemetry, TSE Systems, Inc., Chesterfield, MO, United States) were installed based on stereotaxic coordinates selected from the atlas of Salinas-Zeballos et al. (1986) and adjusted based on the piglet size and neuroanatomical references. Coordinates for each electrode were verified as previously described (Martin et al., 1997a; Martin, 2003; Brambrink et al., 1999). Miniature cranial screws were inserted epidurally through burr holes, and the electrode-epidural screw assembly was securely mounted with low-heat, quick-set acrylic cement. The transmitter was inserted in a nape pocket, and the array antenna was secured to the posterior-most point of the scalp, where it was sutured.

Piglets were intubated and mechanically ventilated using a Datex-Ohmeda Aestiva/5 anesthesia system equipped with a SmartVent ventilator. Each piglet was assessed for normal physiological baseline measurements, including core body temperature (38.0–38.5 °C), mean arterial pressure, heart rate, and blood parameters (glucose, pH, PaCO_2_, PaO_2_, SaO_2_, and hemoglobin). Blood pressures and heart rates were determined by amplifiers. Arterial blood gas and chemistry were obtained using a Radiometer ABL 825 FLEX blood gas analyzer with oximetry and electrolyte modules. They inhaled 10% oxygen for 45 min, lowering the PaO_2_ to ~30 mmHg and SaO_2_ to ~30%, followed by room air for 5 min to slightly reoxygenate tissues. Then, the endotracheal tube was clamped to induce asphyxia (PaO_2_ ~ 15–18 mm Hg and SaO_2_ ~ 3–5%) for 8 min, leading to severe bradycardia (~50 beats per minute) and hypotension (mean arterial blood pressure, MAP, ~25–30 mmHg). Resuscitation was done with 50% oxygen, chest compressions, and epinephrine (100 mcg/kg intravenous). Piglets that failed to regain return of spontaneous circulation within 3 min were excluded. Sham piglets underwent identical anesthesia, surgical preparation, epidural electrode implantation, physiological monitoring, and continuous EEG acquisition as HI piglets but were not subjected to HI or asphyxia. Sham animals received 50% oxygen for 3 min without endotracheal tube clamping or cardiovascular compromise. Two sham groups were included: sham normothermia (Sham-NT) and sham hypothermia (Sham-HT). Sham-NT piglets were maintained at normothermia (38.0–38.5 °C) throughout the protocol. Sham-HT piglets underwent whole-body hypothermia initiated 2 h after the sham procedure, using the same cooling methods, target temperature (34 °C), duration, sedation, neuromuscular blockade, hemodynamic support, and controlled rewarming protocol as HI-HT piglets. This design controlled for the independent effects of anesthesia, surgical instrumentation, continuous EEG monitoring, and HT and rewarming in the absence of hypoxic–ischemic injury.

After resuscitation, whole-body HT was initiated 2 h after resuscitation with ice packs and a water-circulating cooling blanket to achieve a target rectal temperature of 34 °C, mimicking the clinical decrease of 4 °C in HT patients (Rose et al., 1995). The 2-h delay in HT initiation was implemented to model the clinical delay commonly encountered prior to cooling initiation in neonatal patients. Ketamine (10 mg/kg/h intravenously) was initiated 3 h after resuscitation, and nitrous oxide 33% was delivered in a mixture with oxygen 33% and air 33%. Dopamine was administered to maintain MAP above 40 mmHg during the overnight HT or NT protocols while the piglets were anesthetized.

Rewarming to normothermia (NT) commenced in HT piglets 20 h after the protocol began by gradually increasing the blanket water temperature, aligning with the clinical rewarming rate of 0.5 °C/h. The target temperature was set at 38.5 °C. Vecuronium infusion was discontinued 15 h after the insult to allow neuromuscular blockade to dissipate before extubating. Piglets were extubated when they began to breathe voluntarily, regained muscle tone, were responsive to stimuli, and established a tentative upright posture. Then they were returned to cages with milk and overnight supervision.

### Brain harvesting for neuropathology

Piglets survived 2–7 days post-insult. SomnaSol iv was used for euthanasia. All piglets were immediately perfused through a catheter, introduced into the aortic root, with 4% paraformaldehyde after exsanguination with phosphate-buffered saline. The piglet perfusion-fixation protocol was strictly followed as described previously (Primiani et al., 2023). After decapitation, the cranial vault was opened with rongeurs, and the brains were examined for electrode placement and cortical damage, ensuring electrode screws were epidural. The brains were removed from the skull, immersion fixed overnight, and then immersed in 20% glycerol overnight, blocked coronally, and processed for paraffin embedding.

### H&E Neuropathology, immunostaining, imaging, and quantification

Human and piglet paraffin-embedded brain tissue blocks were sectioned at 10 μm using a Microtome (Leica RM2255). Sections were mounted, dried at 40 °C for 16 h, deparaffinized, and rehydrated with ethanol and deionized water. Sections were stained with hematoxylin and eosin (H&E). Microscopic analyses were done blinded to treatment in the anterior primary somatosensory cortex, confirmed by cytology, chemo-architecture, and connectivity as previously described (Primiani et al., 2023; Martin et al., 1997a). Ischemic neurons, identified by eosinophilic cytoplasm and basophilic nuclear condensation, were quantified by profile counting within non-overlapping microscopic fields in the somatosensory cortex using a light microscope at 400X magnification. For immunohistochemistry, antigen retrieval was performed at 95 °C in sodium citrate buffer (pH 6.0) for 20 min. Permeabilization and blocking were achieved using 1X Tris-buffered saline (TBS) containing 0.4% TritonX-100 and 10% normal goat serum (NGS). Tissue sections were incubated overnight at room temperature in a dark, hydrated chamber with antibodies to GFAP (1:200, Millipore MAB360), GLAST (1:1000, Protein Tech 20,785-1-AP), and synaptophysin (1:500, Synaptic Systems, sysy101008) diluted in 1X TBS with 0.1% TritonX-100 and 10% NGS. These primary antibodies have been characterized for specificity in pig brain (Martin et al., 1997b). The secondary antibodies, goat anti-rabbit Alexa 488 (Invitrogen) and goat anti-mouse Texas Red^®^ (Invitrogen), were diluted 1:400 in 1X TBS with 10% NGS and incubated for 2 h at room temperature in the hydrated chamber. The sections were washed in 1X TBS and mounted with Vectasheild with DAPI (Vectashield^®^ H-1800) hardening mounting medium. Confocal imaging was performed using a MICA (Leica) system at 63x magnification. Image processing and quantification were conducted with Fiji. Synaptophysin- and GLAST-positive particles (0.05–3 μm^2^ and contours 0.3–1) and GFAP-positive particles (1 μm^2^ and contour 0.3–1) were quantified. Qualitative neuropathological assessment by H&E staining was used to identify regions of ischemic neuronal injury and to guide region of interest selection for quantitative analyses. Quantitative measures of GLAST, synaptophysin, and GFAP immunoreactivity were chosen to assess astrocyte-mediated glutamate transporter localization, synaptic integrity, and reactive astroglial responses, respectively. These cellular and synaptic markers were selected based on their established roles in excitatory neurotransmission and seizure susceptibility, enabling direct comparison between structural astrocyte-synapse pathology and functional outcomes measured by cEEG, including seizure burden and progression. Where indicated, quantitative neuropathological measures were correlated with EEG-derived seizure metrics to assess structure–function relationships.

### Data acquisition and automatic seizure analysis

Signals were sampled at 250 Hz and stored in an NSS graphics file format using Notocord-HEM^®^. Noise reduction was performed through the preset function. Automatic seizure detection was performed using the seizure detection (SZR) module that flags ictal-like epochs based on user-defined amplitude, frequency, and duration criteria, with a base root mean squared (RMS) three times greater than the baseline and manually inspected to confirm ictal seizure events. Results were compared to previous results reported by clinicians (Primiani et al., 2023), and discrepancies were reconciled by consensus. The same detection parameters were applied uniformly to all recordings across both NT and HT groups to ensure consistent sensitivity. Recordings were exported to European data file (EDF) format for more detailed analysis (Supplementary Figure 4A).

### EEG analysis pipeline with feature extraction and multi-metric seizure classification

To characterize EEG signals and classify seizures (Martin et al., 1997a; Mirowski et al., 2009; Litt and Echauz, n.d.), the analysis pipeline was implemented in Python and developed and debugged in PyCharm (V.2024.3.4. JetBrains) integrated development environment (IDE). Core libraries included MNE-Python (EEG I/O and montage handling), NumPy/SciPy (Signal processing), pandas (tabulation), matplotlib (figures), PyTorch (GPU-accelerated convolution: MPS backend on macOS), and antropy/nolds (entropy and Hurst exponent). ChatGPT-4o (OpenAI) was used to assist with refactoring, logging, and debugging.

Signals were preprocessed with a band-pass filter (1–120 Hz). Channel names and locations were standardized and mapped to a custom montage based on standard human 10–20 scalp EEG channels. Independent component analysis (ICA) was performed to identify and visualize components contributing to seizure-related activity. We used a multi-metric seizure detection method to compute four quantitative biomarkers for each epoch. Power Spectral Density (PSD) features were extracted across six bands: Delta (1–4 Hz), Theta (4–8 Hz), Alpha (8–12 Hz), Beta (12–30 Hz), Slow Gamma (30–60 Hz), and Fast Gamma (70–120 Hz) (Martin et al., 1997a). For each seizure epoch, Spectral Entropy (Shannon entropy) (Bandarabadi et al., 2015; Helakari et al., 2019; Sato et al., 2019; Guerrero-Aranda et al., 2023), RMS Amplitude (Mirowski et al., 2009), Coherence (Akter MostS et al., 2020), and the Hurst Exponent (Schindler et al., 2007; Mielniczuk and Wojdyłło, 2007) were calculated to classify seizure types (Supplementary Figure 4B). Results were compared to previously published results analyzed by clinicians (Primiani et al., 2023).

### Seizure classification criteria

Seizures were classified as generalized or focal based on a majority voting scheme using the extracted features. Thresholds were selected via sensitivity analysis to optimize separation between focal and generalized phenotypes. These numeric cut-point thresholds were empirically optimized against our manually validated ground truth (Primiani et al., 2023) to maximize classification accuracy and were applied uniformly across all animals and treatment conditions. A low-frequency power (delta/fast gamma ratio of >1.5) suggests generalized seizure, lower global synchrony reflecting restricted onset zone (alpha/beta ratio of >1.2) suggests focal seizure; greater signal unpredictability (spectral entropy of > baseline) favors generalized seizure, higher network synchrony (coherence of > baseline) favors generalized seizure; less long-range temporal persistence (Hurst exponent < baseline) favors generalized seizure. A seizure was classified as generalized if ≥3 of the above criteria were met.

### Sample size and statistical analysis

Statistical analysis was conducted using Prism 9.0 (GraphPad). Sample sizes were estimated based on effect sizes reported in previous HI studies with large animals, targeting the detection of a 30% difference in seizure burden with 80% power at *α* = 0.05. Given the large effect sizes observed in EEG-based seizure outcomes, the use of repeated-measures analyses, and ethical and logistical constraints inherent to large neonatal animal models, 4–6 animals per group were deemed sufficient. Individual piglets assigned to different treatments using a randomized design and piglet treatment groups in this study were (Histology/EEG) as follows: sham-normothermia (SH-NT, *n* = 6/4), sham-hypothermia (SH-HT, *n* = 9/6), HI-normothermia (HI-NT, *n* = 6/4), and HI-hypothermia (HI-HT, *n* = 6/4). Seizure progression over time was quantified using the Area Under the Curve (AUC), and group comparisons were performed using mixed-effects analysis to account for repeated measurements within individual piglets and incomplete longitudinal data. Data normality was assessed using the Shapiro–Wilk test. Normal-distributed data were analyzed using an ordinary one-way analysis of variance (ANOVA) with multiple comparison testing or an unpaired two-tailed Welch’s t-test when variances were unequal. Non-normally distributed data were analyzed using Kruskal-Wallis tests followed by Dunn’s multiple comparison test or a Mann–Whitney test, as appropriate. For histological quantification, robust regression combined with the ROUT method was used to identify and exclude statistical outliers, minimizing the influence of biological and technical variability. Correlation analyses were performed using simple linear regression, with regression equations and coefficients of determination (R^2^, goodness of fit) reported to assess directional associations. Confidence intervals for regression parameters were examined but not reported, as small sample sizes yield wide and unstable interval estimates that do not meaningfully improve interpretability. Accordingly, correlation analyses were interpreted descriptively. Statistical significance was defined as *p* < 0.05.

## Results

### Neocortical GLAST localization is pathological in human neonatal HIE

**Figure 1:**
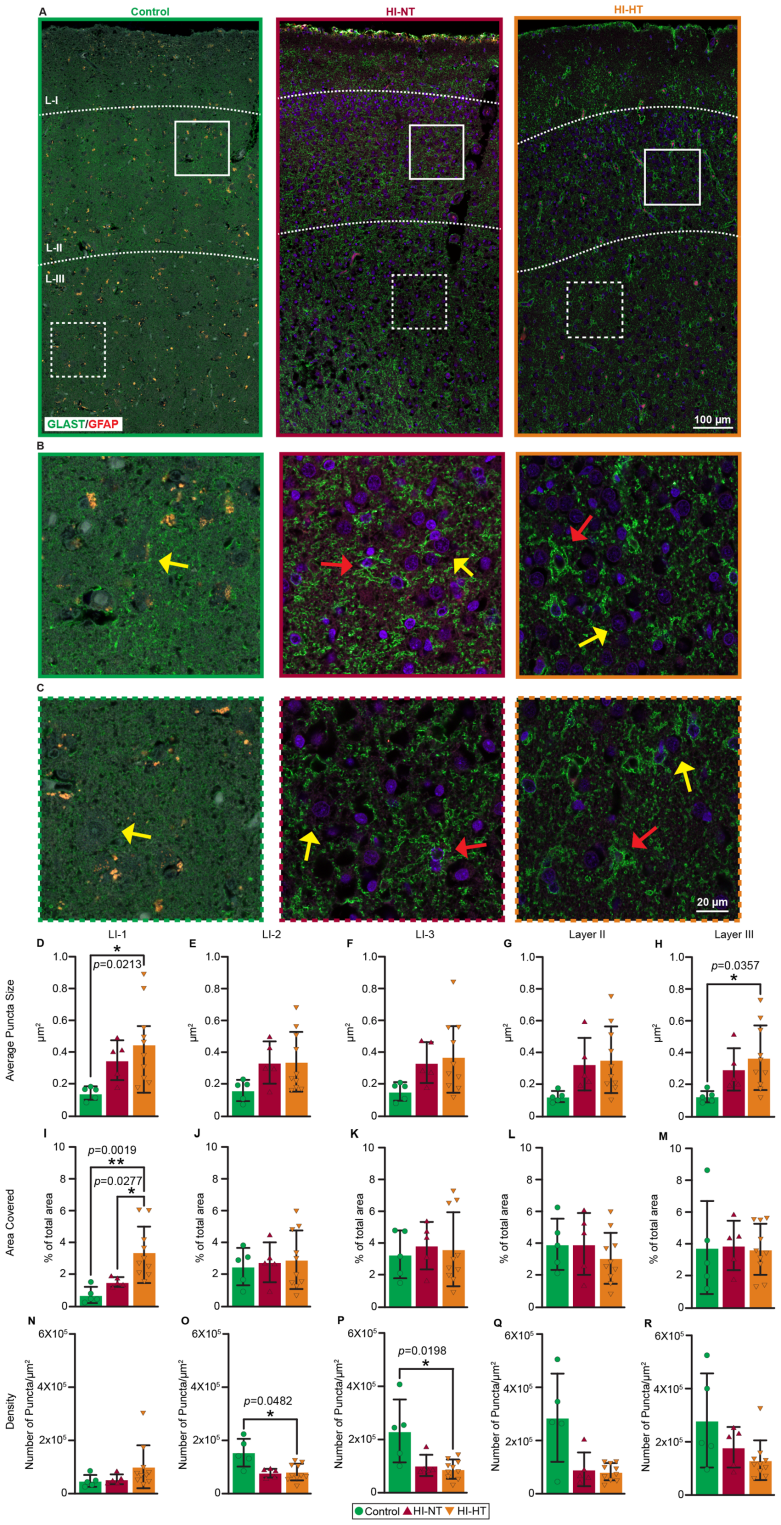
Layer specific abnormal astrocytic GLAST localization in HIE patients. **(A–C)** Localization of GLAST/GFAP in representative images of human neonatal cortex of control, HI-NT, and HI-HT cases. **(A)** Low magnification representative images of human neonatal cortex show GLAST (green) and GFAP (red) in layers I, II, and III. Blue is DAPI nuclear staining. Colored solid or dashed boxes are shown as higher magnification images in **B,C** for each human case. **(B,C)** In layer II **(B)** and layer III **(C)**, GLAST (green) is mostly seen as fine neuropil processes, puncta, and perisomatic labeling (yellow arrows) in control cases. Peri somatic GLAST appears enlarged (red arrows) or aggregated (yellow arrows) in HI-NT and HI-HT cases **(D–R)**. Quantifications of GLAST size, coverage percentage, and density in layers I, II, and III of human cases. **(D–H)** The particle sizes of GLAST were quantified in layers I **(D–F)**, II **(G)**, and III **(H)**. In all layers, the average size of GLAST puncta was increased in HI-NT and HI-HT cases, larger than HI-NT cases. A significant increase in GLAST puncta size was observed in layer I-1 and layer III. **(I–M)** The coverage percentage of GLAST was quantified in layers I **(I–K)**, II **(L)**, and III **(M)**. A significant coverage percentage increase was observed in layer I-1 of HI-HT cases. **(N–R)** The density of GLAST was quantified in layers I **(N–P)**, II **(Q)**, and III **(R)**. Overall reduction of density was observed in all layers, and a significant reduction of density was observed in layers I-2, and 3. Statistical analysis; normality was assessed using the Shapiro–Wilk test. Normal-distributed samples were analyzed using ANOVA with a multiple comparison test. Lognormal-distributed samples were analyzed using the Kruskal-Wallis test followed by a Dunn’s multiple comparison test. *p < 0.05, **p < 0.01, ***p < 0.001, ****p < 0.0001, *p* values are indicated.

We examined GLAST (EAAT1) localization in the neocortex of infant human HIE cases and age-matched non-HIE controls. The HIE cases were divided into those that received in part or in full HT protocols and cases that did not receive HT (Table 1). Double immunofluorescence staining for GLAST/GFAP confirmed astrocytic localization of GLAST (Supplementary Figure 1). We analyzed the cortical sections across layers I-III. Layer I was further subdivided into three sublayers – superficial, middle, and deep – to evaluate spatial changes in astrocytic end feet at the cortical surface (near the glial limitans). Puncta size, fractional coverage, and puncta density were quantified in each sublayer (Figure 1). Across all cortical layers, human HIE cases showed a consistent increase in GLAST puncta size compared to controls, independent of HT treatment. In layer I-1, mean puncta nearly doubled in size (control, − 0.2 μm^2^; HI, −0.4 μm^2^). The increase was significant in HI-HT infants in layer I-1 (*p* = 0.0213). A similar enlargement was detected in layer III (*p* = 0.0213) (Figures 1D,H). Fractional GLAST coverage of neuronal cell bodies (perisomatic) was markedly elevated in the superficial layer I-1; it increased from <1% in controls to 1% in HI-NT (*p* = 0.0019) and > 3% in HI-HT infants (*p* = 0.0277) (Figure 1I). In contrast, GLAST puncta density displayed an inverse pattern. While layer I-1 showed a slight, non-significant increase, the deeper sublayers I-2 and I-3 exhibited approximately 50% reductions relative to controls (*p* = 0.0277 and *p* = 0.0198, respectively) (Figure 1P). A layer-by-layer analysis revealed an overall decrease in GLAST puncta density from layer I through layer III (Figures 1N–R).

### Astrocytic GLAST localization is abnormal in a piglet model of HI and is rescued by HT

**Figure 2:**
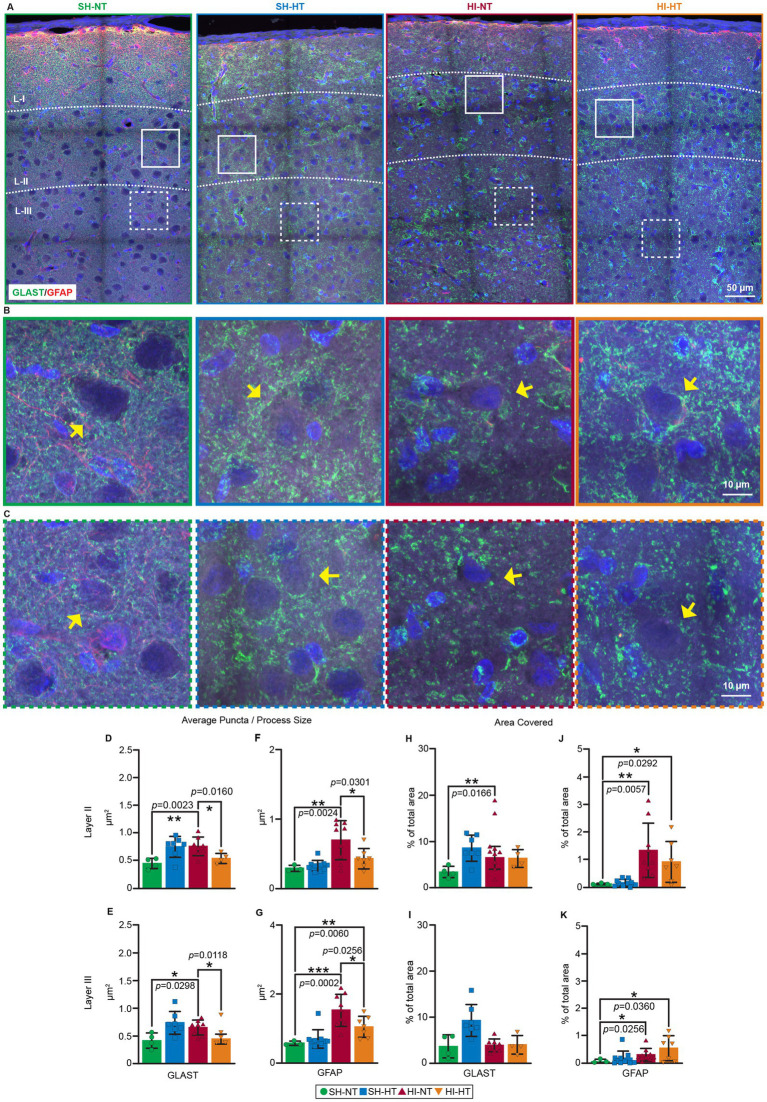
Layer-specific aggregation of GLAST immunoreactivity in the somatosensory cortex of HI-NT piglets is attenuated by HT. **(A–C)** Localization of GLAST/GFAP immunoreactivities in representative images of the somatosensory cortex of sham and HI piglets without and with HT-rewarming treatment. **(A)** Low magnification representative images of somatosensory cortex show GLAST (green) and GFAP (red) immunoreactivities in layers I, II, and III. Blue is DAPI nuclear staining. Colored solid or dashed boxes are shown as higher magnification images in **(B,C)** for each piglet treatment group. **(B,C)** In layer II **(B)** and layer III **(C)**, GLAST immunoreactivity (green) is mostly seen as fine neuropil processes, puncta, and perisomatic labeling (yellow arrows) in sham-HT piglets. Peri-somatic GLAST immunoreactivity appears swollen or aggregated (yellow arrows) in SH-HT and HI-NT piglets. HI-HT had a normalized GLAST immunoreactivity (yellow arrow) pattern. **(D–G)** The particle sizes of GLAST and GFAP immunoreactivities were quantified in layers II **(D,E)** and III **(F,G)**. In layer II and layer II, the average size of GLAST puncta was increased in SH-HT and HI-NT piglets, while puncta size in HI-HT piglets was significantly reduced compared to HI-NT piglets. GFAP immunoreactivity was also significantly increased in HI-HT piglets, and this abnormality was mitigated in HI-HT piglets. **p < 0.001, *p < 0.01, *p* values are indicated. **(H,K)** Percentage of somal area covered by GLAST and GFAP in layer II and layer III. GLAST immunoreactivity increased significantly in layer II **(H)** only in HI-NT piglets. Percent coverage area by GFAP immunoreactivity is increased in both layer II **(J)** and layer III **(K)**. Statistical analysis was done by Welch’s T test to compare each group against the SH-NT group. *p < 0.05, **p < 0.01, ***p < 0.001, ****p < 0.0001, *p* values are indicated.

To determine whether the GLAST abnormalities observed in human neonatal HIE neocortex are mirrored in our piglet model, we examined GLAST localization in the neocortex of HI piglets (Figure 2). In SH-NT piglets, GLAST displayed fine, evenly distributed puncta in cortical layer I neuropil and smooth, subtle peri-neuronal elongation observed in layers II/III (Figures 2B,C), consistent with normal astrocytic GLAST puncta morphology (fine, delicate, wispy, dispersed, and smoothly sidled). In SH-HT piglets, GLAST puncta appeared slightly larger and more irregular in shape (Figures 2B,C), suggesting subtle structural remodeling induced by HT alone, independent of the anesthesia effect (Figures 2A–C). Following HI, the GLAST organization was severely disrupted in HI-NT piglets. Immunolabeling revealed process fragmentation, reduced perisomatic coverage, and loss of fine neuropil puncta (Figures 2B,C) resembling the GLAST disorganization seen in human HIE cortex. In contrast, GLAST localization in HI-HT piglets remained relatively normal, showing preserved peri somatic and extracellular distribution (Figures 2B,C). Quantitative analysis confirmed a significant increase in GLAST puncta size (layer II, *p* = 0.0023; layer III, *p* = 0.0298) in layers II/III of HI-NT piglets compared to SH-NT animals, and these changes were protected by HT in HI-HT piglets (layer II, *p* = 0.0160; layer III, *p* = 0.0118) in layer II/III (Figures 2D,H). These quantifications were supported by an increase in fractional area coverage (layer II, *p* = 0.0166) in layers II/III (Figure 2E) of HI-NT piglets compared to SH-NT piglets. Quantification of GLAST density in layers I/II/III of HI-NT piglets showed no changes compared to SH-NT piglets. GLAST Co-immunostaining for GFAP supported these findings, showing enlarged but structurally preserved astrocytic processes in the same cortical regions (Figures 2F,G). Process size increase was observed in layer II/III in HI-NT piglets compared to SH-NT piglets (layer II, *p* = 0.0024; layer III, *p* = 0.0002), and HT effectively protected these changes to a SH-NT comparable level (layer II, Figure 2F, *p* = 0.0301; layer III, Figure 2G, *p* = 0.0256). HT effectively preserved GLAST integrity and structure, and prevented these pathological changes, and GLAST coverage was selectively increased in layer II of HI-HT piglets (Figure 2H, *p* = 0.0166).

### HI piglets have robust cEEG identifiable seizures and laminar selective neocortical neuropathology

**Figure 3:**
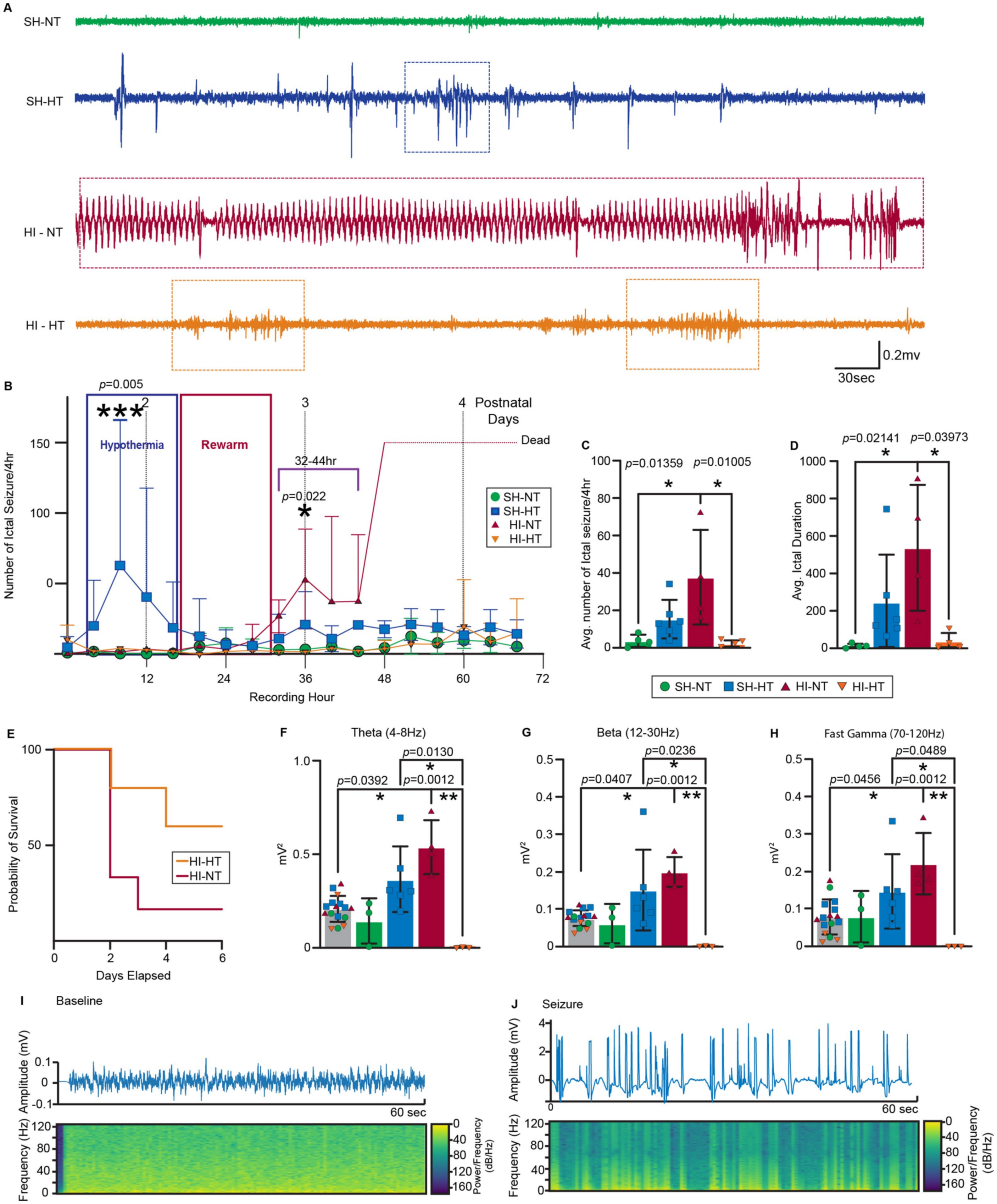
HI piglets have cEEG-identified seizures and layer-specific cortical neuropathology suppressed by HT. **(A)** Representative 30-min EEG recordings from each experimental group. Seizure events are marked with matching color boxes. **(B)** Time trace of average number of ictal seizures during EEG recording period through HT (0 h–12 h), rewarming (16 h–32 h), and recovery period (32 h–68 h). EEG was recorded starting generally on postnatal day 2 with continuous telemetry through postnatal day 5. For statistical analysis, the area under the curve was calculated, and mixed-effects analysis with multiple comparison test was done. Significance ***p < 0.001, *p < 0.05. **(C,D)** Quantitative analysis (cEEG timeframe hours 32-44) of the average number of ictal events per 4 h **(C)** and average duration of ictal seizure events **(D)**. Statistical analysis was done with a Shapiro–Wilk normality test, and one-way ANOVA with multiple comparison test followed by Tukey’s multiple comparisons test, or a Kruskal-Wallis test followed by Dunn’s multiple comparisons test with 95% confidence. *p < 0.05, *p* values indicated. **(E)** Kaplan–Meier survival curves for HI-NT and HI-HT piglets. HI-NT piglets had shorter survivals than HI-HT piglets. **(F–H)** Power spectrum density analysis of ictal seizure event voltages (mV^2^) for normalized (PSD compared against baseline) theta wave (**F**, 4–8 Hz), beta wave (12–30 Hz), and fast gamma wave (70–120 Hz). Statistical analysis was done with a Shapiro–Wilk normality test, followed by Tukey’s multiple comparisons test or a Kruskal-Wallis test, followed by Dunn’s multiple comparisons test with 95% confidence. *p < 0.05. *p-*values indicated. **(I,J)** Representative piglet EEG signals with spectrograms for baseline **(I)** and seizure **(J)**.

Because HT preserved GLAST localization and astrocytic structure in the HI-HT piglets, we next examined whether this preservation translated into functional neuroprotection by reducing seizure activity. We continuously recorded EEGs throughout the cooling, rewarming, and recovery phases. cEEG traces from each experimental group were first visually inspected (Figure 3A). The SZR module, an automated seizure detection function integrated into the Notocord-HEM^®^ platform, with a base root mean squared (RMS) three times greater than the baseline. Flagged events were manually inspected to confirm ictal seizure events. Final results were compared to previous results reported by clinicians (Primiani et al., 2023), and discrepancies were reconciled by consensus (Supplementary Figure 5A). Cleared events were binned into 4-h intervals and plotted over time to assess temporal evolution across treatment groups. Interestingly, some SH-HT piglets exhibited brief seizure-like discharges that occurred from 0 to 24 h, with peak activity at ~8 h (Figure 3B, *p* = 0.005) during the HT phase (Figure 3B). These events showed considerable inter-experimental group variability, suggesting that HT alone or in combination with an anesthetic, even in the absence of HI insult, can induce spontaneous recurrent seizures (SRS) in some piglets. In the HI-NT piglets, no seizure activity was observed during the first 24 h post-injury (during most of the HT phase). However, seizures emerged at ~28 h and peaked at 36 h post-insult (Figure 3B, *p* = 0.022). These seizures were severe and frequently fatal, with over 50% of HI-NT piglets needing to be euthanized within 48 h post-insult (Figure 1E). In contrast, EEG recordings from HI-HT piglets showed no seizure activity until 52 h post-injury, thus seizure onset was delayed significantly, and seizure frequency remained low and generally not life-threatening (Figures 3B,E). The mixed effect analysis of seizure progression revealed that ictal seizure-like event frequency from 32 h to 44 h was significantly (Figure 3C, *p* = 0.01359) high in HI-NT piglets (Figure 3C) and duration of ictal seizure-like events was significantly prolonged (Figure 3D, *p* = 0.02141), accompanied by high inter-animal variability (Figure 3D). HI-HT piglets exhibited significantly fewer to no ictal events (Figure 3C, *p* = 0.01005), and those observed had markedly shorter duration with low inter-animal variability (Figure 3D, *p* = 0.03973). These findings suggest that HT effectively delays seizure onset and reduces seizure burden in neonatal HI piglets.

We analyzed the PSD across canonical frequency bands (Figures 3F–J). Notably, theta (4–8 Hz, Figure 3F, *p* = 0.0392), beta (12–30 Hz, Figure 3G, *p* = 0.0407), and fast gamma (70–120 Hz, Figure 3H, *p* = 0.0456) band activities were markedly elevated in HI-NT piglets compared to the baseline (Figures 3F–H), indicating heightened network excitability after HI. Although PSDs of these bands were heightened in SH-HT piglets, the changes were not statistically significant. In contrast, PSDs in the HI-HT piglets were significantly reduced compared to baseline levels across all frequency bands (Theta, Figure 3F, *p* = 0.00012; Beta, Figure 3G, *p* = 0.00012; Fast Gamma, Figure 3H, *p* = 0.00012) (Supplementary Figures 6A–C), suggesting a profound suppression of neural network activity, defined as the dynamic putative synaptic signals across interconnected neuronal populations.

H&E staining was used to quantify neuronal injury in the somatosensory cortex (Supplementary Figure 4A) near where the electrodes were implanted, so the cEEG metrics can be related to the presence of ischemic neurons and GLAST immunoreactivity. HI piglets exhibited characteristic ischemic neuronal degeneration, including pyramidal neuron necrosis indicated by somal eosinophilia and attrition, nuclear pyknosis, neuropil vacuolation (Supplementary Figure 4B, red arrows), and swelling of peri somatic space in both HI groups (Supplementary Figure 4B, yellow arrows). Quantitative analysis revealed a marked increase in ischemic-necrotic neurons in layer III (Supplementary Figure 4E, *p* = 0.0001), with trends for increases in layer I and layer II (Supplementary Figures 4C,D). HT significantly attenuated neuronal necrosis, particularly in layer III, consistent with selective larminar neuroprotection (Supplementary Figure 4E).

### Cortical depth dependent correlations between GLAST expression pattern and seizure activity are revealed under HT

**Figure 4:**
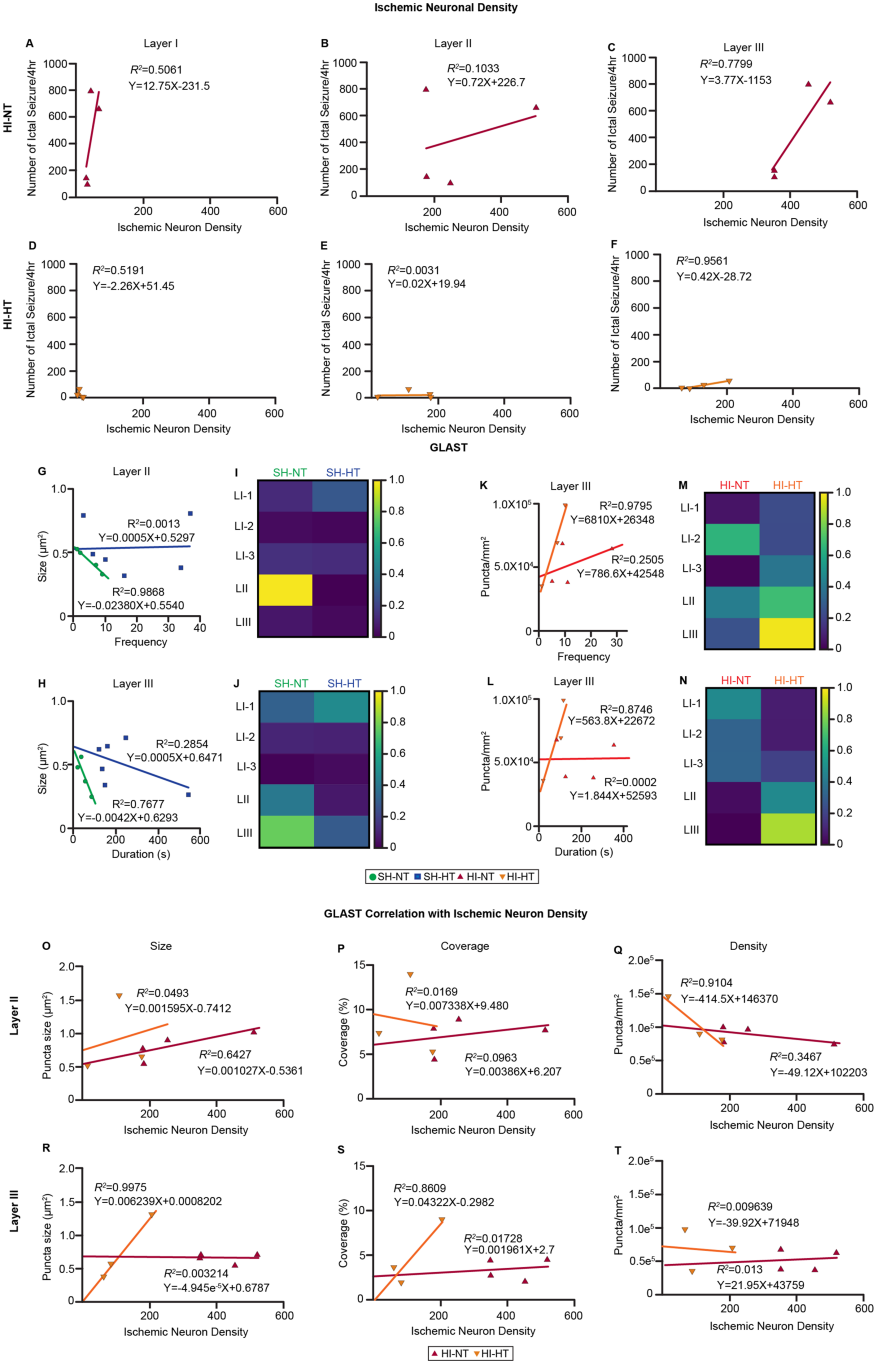
Layer-specific neuropathology correlates with spontaneous recurrent seizures metrics after HI in piglets. **(A–F)** Simple linear regression correlation analysis of cortical layer-specific ischemic neuron density and seizure frequency in HI piglets. **(A–C)** Cortical layer-specific ischemic neuron density correlations with ictal seizure frequency in HI-NT piglets. HI-NT piglets had a slight positive correlation with a steep slope in layer I (R^2^ = 0.5061), low correlation with a relatively low slope in layer II (R^2^ = 0.1033), and a stronger positive correlation with a medium slope (R^2^ = 0.7799) in layer III. **(D–F)** Cortical layer-specific ischemic neuron density correlations with ictal seizure frequency in HI-HT piglets. HI-HT piglets had a slight positive correlation in layer I (R^2^ = 0.5191), like HI-NT piglets, a very low correlation with negligible slope in layer II (R^2^ = 0.0031), and a stronger positive correlation (R^2^ = 0.9561) in layer III. **(G,H)** Cortical layer-specific GLAST puncta size correlations with ictal seizure in SH piglets. SH-NT piglets had a strong inverse correlation with GLAST puncta size and seizure frequency in layer II (**G**, R^2^ = 0.9868) and duration in layer III (**H**, R^2^ = 0.7677). **(I,J)** Heatmap of R^2^ values of SH piglets **(I)** shows layer II specific correlation of GLAST puncta size with seizure frequency, and **(J)** shows layer III specific correlation of GLAST puncta size with seizure duration, and with a slight increasing trend in R^2^ value peaking at layer III. **(K,L)** Cortical layer-specific GLAST density correlation with ictal seizure in HI piglets. HI-HT piglets had a strong positive correlation with GLAST puncta size and seizure frequency in layer III (**G**, R^2^ = 0.9795) and duration in layer III (**H**, R^2^ = 0.8746). **(M,N)** Heatmap of R^2^ values of HI piglets **(M)** shows the layer III specific correlation of GLAST density with seizure frequency, and **(N)** shows the layer III specific correlation of GLAST density with seizure duration. Duration and frequency correlation showed an increasing trend in value, peaking at layer III. **(O–T)** Simple linear regression correlation of cortical layer-specific ischemic neuron density with GLAST structural metrics of HI piglets. **(O–Q)** In cortical layer II, ischemic neuron density of HI-NT piglets shows positive correlation with size **(O)** and coverage **(P)** of GLAST when density **(Q)** shows inverse correlation. HI-HT piglets showed positive correlations with size **(O)** and inverse correlations with coverage **(P)** and density **(Q)**. **(R–T)** In cortical layer III, ischemic neuron density of HI-NT piglets does not show a notable correlation, but HI-HT piglets show a strong positive correlation with size **(R)** and coverage **(S)**. R^2^ values and response equations are as indicated in the graph.

**Figure 5:**
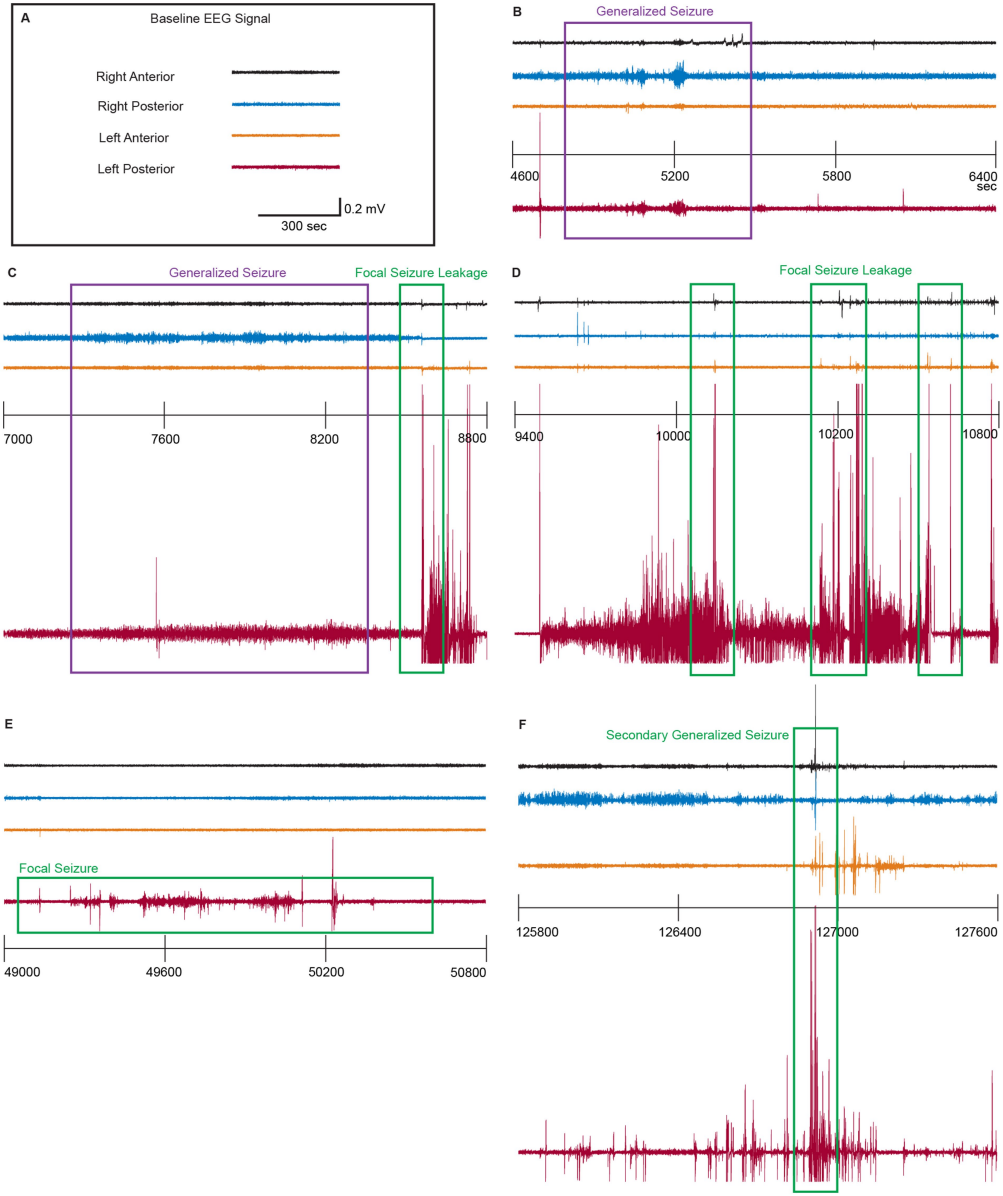
Complex cEEG seizure patterns are seen in HI-HT piglets at days 4 and 5. Representative cEEG recordings (30-min segments) from 4 epidural electrodes at different times after HT and rewarming from the left and right somatosensory cortex of an HI-HT piglet. **(A)** Baseline EEG signal. **(B)** Around 5,200 s into the 4th day after the HI and treatment, prominent ictal-like bursts with modest postictal depression were detected in both right (blue) and left (red) posterior somatosensory cortices (purple box). Weaker ictal-like bursts, suspected as generalized seizures, were recorded from both right (black) and left anterior (orange) somatosensory cortices. **(C)** Approximately 2 h later than the recordings shown in **(B)**, dense, sharp spikes were detected in both right (blue) and left (red) posterior somatosensory cortices from 7,500 s to 8,200 s (purple box) and were suspected as generalized seizures. Then, 600 s later, high-amplitude spike bursts emerged (green box) but only in the left posterior somatosensory cortex. Coincident short, sharp spikes were recorded from other areas, at the time the maximum amplitude was recorded from the left posterior (red). **(D)** About 20 min after recording in panel **C**, sharp, low-amplitude spikes in other areas (black, blue, orange) coincided with maximum magnitude spikes recorded from the left posterior (red) somatosensory cortex (green boxes) and were suspected as a focal seizure. **(E)** At about 13.5 h into day 4 post-HI, suspected focal seizures were seen as ictal-like bursts in the left posterior (red) somatosensory cortex (green box). **(F)** About 10 h into day 5 after HI, suspected secondary generalized seizures possibly triggered by prior focal seizures were detected as sharp spikes in all areas, coinciding with the maximum magnitude spike recorded from the left posterior (red) somatosensory cortex. Amplitudes of spikes recorded from other areas also increased compared to previously observed similar incidents (green box).

To determine whether the preservation of astrocytic GLAST was functionally linked to reduced seizure activity, we performed correlation analyses between neuropathological markers and electrophysiological metrics. Specifically, we analyzed relationships among ischemic neuronal density, GLAST puncta size or density, and ictal seizure frequency and duration across cortical layers I/II/III in all piglet groups (Figures 4A–F; Supplementary Figure 7). In HI-NT piglets, ischemic neuronal density showed positive correlation with seizure frequency across all cortical layers, with the strongest association in layer III (Figures 4A–C, R^2^ = 0.7799). In contrast, HI-HT piglets displayed correlations indicating cortical depth-dependent reorganization. A weak inverse relationship in layer I (Figure 4D, R^2^ = 0.5191), no correlation in layer II (Figure 4E), and a strong positive correlation in layer III (Figure 5F, R^2^ = 0.9561). We next examined the relationship between GLAST structural metrics and seizure parameters (Figures 4G–N). In SH-NT piglets, seizure frequency inversely correlated with GLAST puncta size in layer II (Figure 4G, R^2^ = 0.9868), and seizure duration inversely correlated with puncta size in layer III (Figure 4H, R^2^ = 0.7677), and the heatmap of R^2^ values showed no specific trend through layers I/II/III (Figures 5I,J), suggesting that GLAST puncta size in specific layer is linked to a specific character of spontaneous cortical excitability under physiological conditions. No significant correlations were observed in SH-HT animals (Figures 5G–J). In HI-NT piglets, correlations between seizure burden and GLAST metrics were weak and inconsistent (Figures 5K–N); in contrast, HI-HT piglets exhibited robust positive correlations between GLAST density and both seizure frequency (Figure 5K, R^2^ = 0.9795) and seizure duration (Figure 5L, R^2^ = 0.8746). Heatmap analyses of R^2^ values demonstrated a progressive strengthening of correlations with cortical depth, peaking in layer III (Figures 5M,N). We then examined the relationship between GLAST structural metrics and ischemic neuron density (Figures 4O–T). In HI-NT piglets, only the cortical layer II ischemic density showed a notable correlation with GLAST structural metrics (Figures 4O–Q). In cortical layer II, ischemic neuron density had a positive correlation with GLAST puncta size (Figure 4O, R^2^ = 0.6427), a positive albeit weak correlation with coverage (Figure 4P, R^2^ = 0.0963), inverse correlation with density (Figure 4Q, R^2^ = 0.3467). In HI-HT piglets, both cortical layer II and III showed notable correlation with specific GLAST structural metrics (Figures 4Q–S). In layer II, ischemic neuron density had shown a strong inverse correlation with GLAST density (Figure 4Q, R^2^ = 0.9104), and in layer III, ischemic neuronal density showed a strong positive correlation with GLAST puncta size (Figure 4R, R^2^ = 0.9975) and coverage (Figure 4S, R^2^ = 0.8609). This analysis additionally supports cortical layer-specific GLAST functional correlation in neuropathology and seizure metrics.

### HI induces complex seizure patterns

While we visually inspected individual EEG traces to confirm automatically detected ictal-like seizure events, we discovered possibly multiple distinct seizure types co-occurring (Figure 5) in each piglet. Importantly, the baseline EEG was similar among the channels in different treatment groups (Figure 5A). We identified at least two distinct types of seizures recorded from piglets. For instance, approximately 2 days after the initial HI insult and about 5,200 s into the day, low-amplitude ictal-like bursts were detected in left and right posterior somatosensory cortices (Figure 5B, purple box), suggesting early signs of coherent bilateral network activity and the potential onset of a generalized seizure. Between 7,000 and 8,200 s, the amplitude of EEG activity gradually increased in both left and right posterior somatosensory cortices (Figure 5C, purple box), culminating in high-amplitude bursts followed by post-ictal depression, dominantly in the left posterior cortex alone (Figure 5C, green box). During these high-amplitude discharges seen in the left posterior cortex, transient “spike blips” or “leakage spikes” were observed in other cortical regions, indicating partial propagation or subthreshold synchronization (Figure 5C, green box). Subsequently, after periods of normal EEG activity, sudden spiking reappeared in the left posterior somatosensory cortex (Figure 5D), again accompanied by synchronous “leakage spikes” in adjacent regions (Figure 5D, green box). At approximately 49,000 s into day 2, focal seizure activity was detected exclusively in the left posterior somatosensory cortex (Figure 5E, green box). As seizures evolved, these focal discharges progressed into synchronized high-amplitude (note voltage) spikes across multiple channels, culminating in secondary generalized seizure activity by approximately 127,000 s now into day 3 after HI (Figure 5F, green box).

### Focal and generalized seizure exhibit distinct PSD signatures

**Figure 6:**
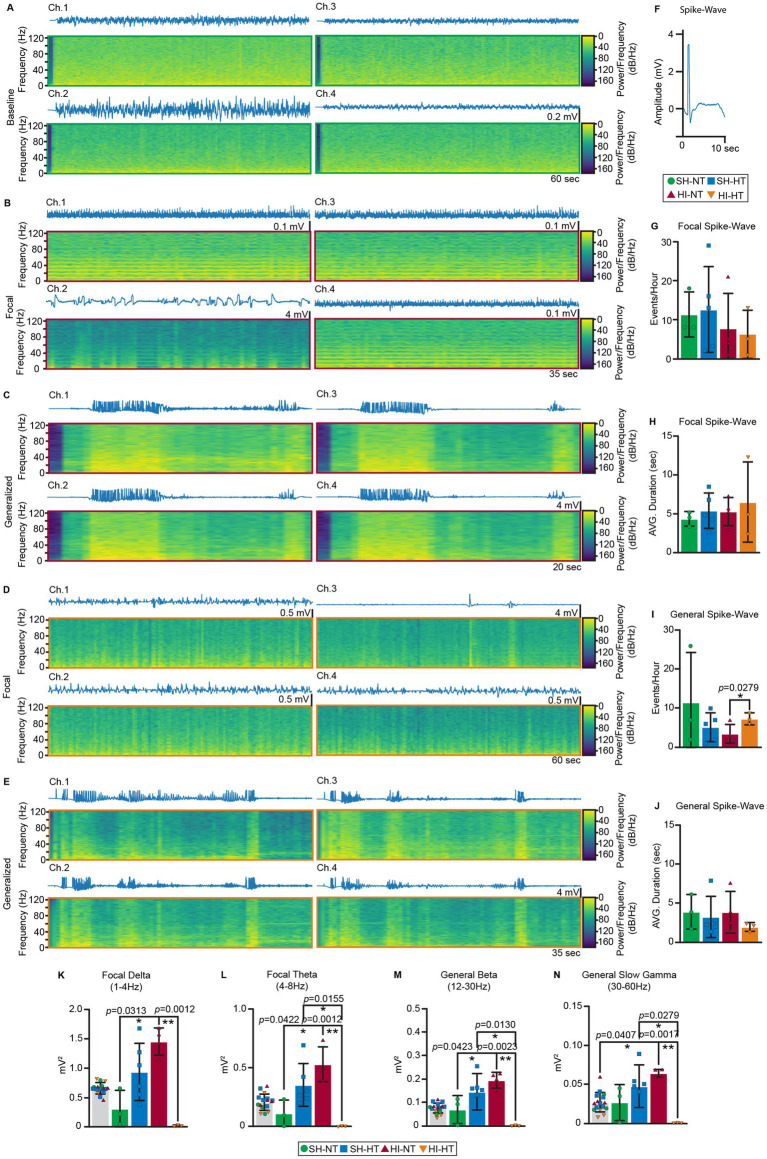
Spike–wave form analysis reveals a distinct PSD pattern in focal vs. generalized seizure. **(A–F)** Representative spike–wave forms derived from cEEG signals and spectrograms from each of the 4 epidural recording electrodes on somatosensory cortex as classifiers of seizures in neonatal piglet HI at NT and with HT and rewarming or with sham procedures. dB spectral power refers to the power spectral density. **(A)** Baseline EEG-spectrogram from SH-NT piglet. **(B)** EEG-spectrogram of focal spike–wave form from HI-NT piglet. **(C)** EEG-spectrogram of putative generalized spike–wave form from HI-NT piglet. **(D)** EEG-spectrogram indicative of focal spike–wave form from HI-HT piglet. **(E)** EEG-spectrogram of putative generalized spike–wave form from HI-HT piglet. **(F)** Typical spike–wave form used to classify seizures. **(G–J)** Graphs of quantitative analysis of focal spike–wave events or generalized spike–wave events by represented as the number of events per hour or average duration of spike–wave events. Piglet treatment groups are color-coded. **(G)** Number of focal spike–wave events per hour. **(H)** Average duration of focal spike–wave events. **(I)** Number of generalized spike–wave events per hour. *p < 0.05, *p* value indicated. **(J)** Average duration of generalized spike–wave events. Statistical analysis was done with a Shapiro–Wilk normality test, followed by a Wilcox test with a 95% confidence level. **(K–N)** PSD analysis of focal or generalized spike–wave events. Piglet treatment groups are color-coded. Gary bar represents the invariant average baseline of all treatment groups. **(K)** Normalized PSD of delta (14 Hz) wave from focal spike–wave events. **(L)** Normalized PSD of theta (4–8 Hz) wave from focal spike–wave events. **(M)** Normalized PSD of beta (1,230 Hz) wave from generalized spike–wave events. **(N)** Normalized PSD of slow gamma (3,060 Hz) wave from generalized spike–wave events. Statistical analysis: normality was assessed using the Shapiro–Wilk test. Normal-distributed samples were analyzed using an ANOVA with a multiple comparison test or an unpaired two-tailed Welch’s test. Lognormal-distributed samples were analyzed using the Kruskal-Wallis test followed by a Dunn’s multiple comparison test or a Mann–Whitney test with 95% confidence. *p < 0.05, **p < 0.01, ***p < 0.001, ****p < 0.0001, *p* values are indicated.

Given the diversity of observed seizure patterns and the absence of baseline EEG changes in the four different channels (Figure 6A), we developed an automated classification program to distinguish between seizure types. The program detects spike–waveform morphology (Figure 6F) with quantitative signal parameters including PSD ratio, entropy, coherence, and Hurst exponent. Event classification was performed using a majority voting algorithm (Supplementary Figure 5B), resulting in three distinct categories: baseline, focal seizure, and generalized seizures. Focal spike–wave events were defined by activity confined to a single EEG channel (Figures 6B,D), whereas generalized spike–wave events were simultaneously detected across multiple channels (Figures 6B,E). No significant difference in the number of focal seizure events was seen among experimental groups, though high inter-animal variability was observed (Figure 6G). These focal events typically lasted 5 s on average, though HI-HT piglets showed high inter-animal variability (Figure 6H). In contrast, generalized seizure events were significantly more frequent in HI-HT piglets (Figure 6I, *p* = 0.0279). However, it is critical to contextualize this finding regarding treatment. While more frequent, these generalized events in the HT group were significantly shorter (~3 s vs. ~ 5 s) and possessed significantly lower spectral power across all frequency bands compared to the high-amplitude ictal burdens seen in NT animals (Figures 6J–N). Thus, HT does not worsen seizure burden; rather, it shifts the seizure landscape from prolonged, high-intensity focal ictal events toward brief, spectrally dampened discharges that meet the generalized classification criteria solely due to their synchronous detection across channels. While other groups had an average duration of approximately 4 s with high inter-animal variability, HI-HT piglets exhibited a consistent duration of approximately average of 3 s with minimal inter-animal variability (Figure 6J). PSD analysis revealed that focal spike–wave events were associated with increased power in the delta and theta frequency bands (1–8 Hz) (Figures 6K,L), while generalized events were characterized by elevated beta (12–30 Hz) and slow gamma (30–60 Hz) activity (Figures 6K–M). Notably, PSD values in HI-HT piglets were significantly reduced across all frequency bands as seen before (Figures 3F–H; Supplementary Figures 6A–C, 8A–H), indicating a global suppression of neural activity potentially attributable to HT. Thus, while generalized event counts were higher in HI-HT, these events represent brief, spectrally suppressed synchronization rather than the severe, high-amplitude ictal burdens observed in NT piglets.

### HT does not protect against cortical synaptic vesicle depletion in HI piglets

**Figure 7:**
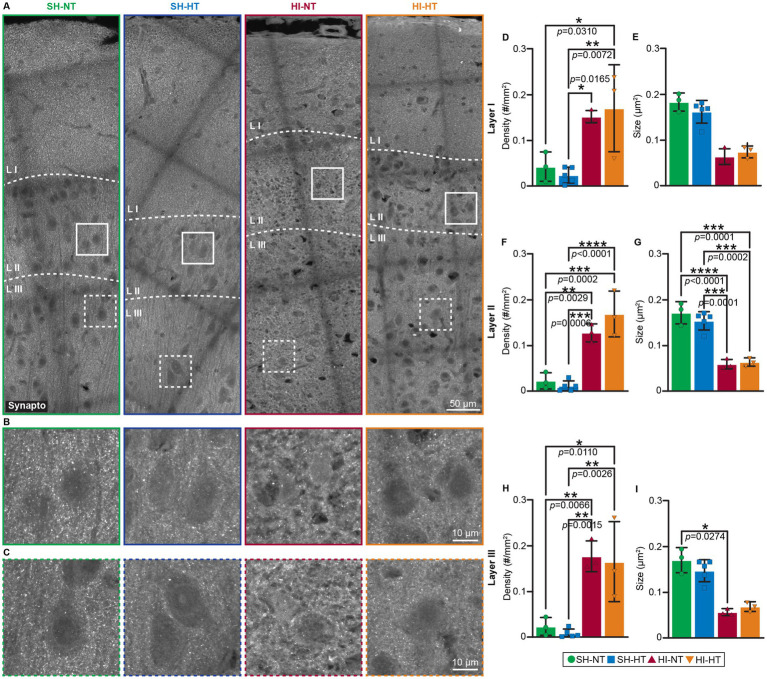
Neocortical synaptophysin immunoreactivity is aberrant in HI-NT piglets and is insensitive to HT. **(A–C)** Localization of synaptophysin immunoreactivity in representative images of the somatosensory cortex of sham and HI piglets without and with HT-rewarming treatment. **(A)** Low magnification representative images of the somatosensory cortex show synaptophysin immunoreactivity in layers I, II, and III. Colored solid or dashed boxes are shown as higher magnification images in **(B)** and **(C)** for each piglet treatment group. **(B,C)** In layer II **(B)** and layer III **(C)**, synaptophysin immunoreactivity is mostly seen as fine neuropil and perisomatic puncta in sham-HT piglets. Synaptophysin immunoreactivity was quantified as the density of particles in layers I, II, and III **(D,F,H)** and as particle size in each layer **(E,G,I)**. Synaptophysin particle number increased in each layer in HI piglets, but size was significantly decreased in layer II in both HI groups and in layer II in the HI-NT group. Statistical analysis was done with a Shapiro–Wilk normality test, followed by Tukey’s multiple comparisons test or a Kruskal-Wallis test, followed by Dunn’s multiple comparisons test with 95% confidence. *p < 0.05, **p < 0.01, ***p < 0.001, ****p < 0.0001, *p* values are indicated.

Synaptic integrity following HI injury and HT treatment was indirectly assessed by immunostaining for synaptophysin (Figure 7), a presynaptic vesicle marker that identifies global presynaptic terminals and is independent of neurotransmitter type (excitatory and inhibitory). Quantitative analysis of fluorescent puncta density and size in layers I, II, and III of the somatosensory cortex of piglets showed significantly increased densities in layers I, II, and III in HI-NT and HI-HT piglets (Figures 7D,F,H). However, qualitative analysis showed highly selective loss of perisomatic presynaptic terminals in HT treated piglets (Figures 7B,C) compared to sham piglets. Significant reductions in synaptophysin-positive puncta size were seen in layers II and III, with only a trend being seen in layer I (Figures 7E,G,I). This synaptic vesicle alteration was observed in both HI-NT and HI-HT piglets (Figures 7D–I), with the most prominent size change occurring in cortical layer II (Figures 7F,G), supporting selective loss of perisomatic presynaptic terminals (Figures 7B,C).

## Discussion

In this study, we used human clinical postmortem HIE brains as a platform to instruct on possible mechanisms of brain injury and epileptogenesis in a translational large animal model of HI using piglets with cEEG monitoring. We found changes in GLAST to be a common abnormality in clinical and experimental HIE. This finding validated our piglet model. We then extensively interrogated EEG changes in HI piglets with or without HT treatment and identified important relationships between GLAST pathology, neurodegeneration, and seizures.

### Human neonatal HIE shows layer-specific GLAST remodeling

Our analysis of human autopsy brains identified a robust astrocytic phenotype in neonatal HIE (Figure 1). Because our optimally prepared piglets had similar changes, we are confident that the human brain findings are not artifact-related to agonal state or postmortem changes. Enlarged GLAST puncta and reduced puncta density were seen in the neocortex of HI cases relative to non-HIE controls. Across layers I–III, GLAST puncta were enlarged, with significant effects in layer I-1 and layer III (Figure 1D, layer I, *p* = 0.0213; Figure 1H, layer III, *p* = 0.0357) of HI-HT patients. Fractional GLAST coverage around neuronal somata was elevated in superficial layer I-1 (Figure 1I, from <1% in controls to ~1% in HI-NT, *p* = 0.0019, and >3% in HI-HT patients, *p* = 0.0277) while GLAST puncta density decreased by ~50% in I-2 (Figure 1O, *p* = 0.0482) and I-3 (Figure 1P, *p* = 0.0198) in HI-HT patients and declined across layers I to III. These combined changes—superficial enlargement and deeper loss— might signify disrupted glutamate clearance microdomains, such as expanded superficial end feet at the glial limitans and perisomatic coverage, with sparser transporter presence at neocortical depths where recurrent excitatory networks reside.

These findings have limitations because they are morphological. Astrocytic glutamate transporters such as GLAST are essential for the rapid removal of extracellular glutamate at the synapse (Moreira et al., 2022; Cui et al., 2024). Under physiological conditions, astrocytes tightly buffer glutamate released from excitatory neurons (Storck et al., 1992; Rothstein et al., 1994), preventing spillover and excitotoxic activation of NMDA/AMPA receptors on neighboring cells (Li et al., 2025; Mahmoud et al., 2019). Dysregulation of these transporters leads to elevated extracellular glutamate, prolonged receptor activation, and increased neuronal and oligodendrocyte depolarization, which can enhance the excitability of cells and axons, lower the seizure threshold, and facilitate synchronous network firing (Cui et al., 2024; Peterson and Binder, 2020). GLAST microdomain disruption, characterized by enlarged superficial puncta and deeper layer loss, may specifically impair glutamate clearance in laminae that integrate corticocortical excitation, thereby predisposing to focal and secondary generalized seizures. Structural preservation of GLAST microdomains by HT could restore glutamate homeostasis, stabilize synaptic transmission, and suppress hyperexcitability. These transporter dynamics form a working cellular model linking astrocyte dysfunction to network hyperexcitability in neonatal HIE (Li et al., 2025; Pajarillo et al., 2019). However, it is uncertain if the GLAST puncta enlargement signifies a pathological process involving protein aggregation and loss of glutamate transporter function or a compensatory hypertrophy process involving GLAST clustering in an attempt to enhance glutamate transport. The persistence of GLAST enlargement in some HT-treated human cases could attest to a positive compensatory effect, or it might reflect severe terminal injury, or a postmortem confounder, rather than a failure of the mechanism. This pattern nevertheless provided the cellular signature we interrogated in our piglet model as it related to epileptogenesis.

### The neonatal piglet paradigm reproduces human-like laminar vulnerability and ictal activity

We have previously reported impaired glutamate uptake in the neocortex following HI in piglets (Martin, 2003), suggesting functional transporter dysfunction. Here, by analyzing the cortical distribution of GLAST in HI piglets (Figure 2) with cEEG-confirmed seizures (Figure 3), we sought to identify the possible structural basis for neonatal seizures. Our animal model recapitulated the neuropathologic and electrophysiologic hallmarks of human HIE. In piglet, the highest ischemic-necrotic neuronal burden was in layer III (Supplementary Figure 4E, *p* = 0.0001), with trends in layers I and II, precisely where the human neocortex integrates corticocortical signaling (Miyashita, 2024). HT conferred laminar-selective neuroprotection by reducing layer III necrosis, while neuropil vacuolation persisted. Continuous 4-channel epidural cEEG captured spontaneous ictal patterns in all HI piglets, with rare cooling-associated events in SH-HT animals, demonstrating that the analysis detects both HI–driven and temperature/anesthesia-modulated excitability. HT favored survival over NT in HI piglets, confirming the whole-body physiological benefit seen clinically.

### HT preserves GLAST organization and dampens astrocytic reactivity in layers II/III

GLAST abnormalities in HI-NT piglets mirrored the human HIE profile. In HI-NT, we observed fragmented processes, perisomatic aggregation, and loss of fine neuropil puncta in layers II/III of the neocortex (Figure 2). Quantitatively, GLAST puncta size increased in layer II (Figure 2D, *p* = 0.0023) and layer III (Figure 2E, *p* = 0.0298) in HI-NT vs. SH-NT, with fractional area coverage also higher in layer II (Figure 2H, *p* = 0.0166) and layer III (Figure 2I, *p* = 0.0256). HT normalized GLAST puncta size to levels seen in SH-NT piglets. The puncta size decreased (Figure 2D, layer II *p* = 0.0160; Figure 2E, layer III *p* = 0.0118; HI-NT vs. HI-HT). Notably, GLAST coverage was selectively increased in layer III under HT (Figure 2I, *p* = 0.0360) with trends in increased density (Supplementary Figure 3), consistent with adaptive reinforcement of glutamate buffering at the lamina most vulnerable to injury and seizures. GLAST density across layers I–III did not significantly differ between SH-NT and HI-NT, and HT induced only a slight, non-significant density rise, indicating that neuroprotection is driven more by microdomain reorganization than gross abundance (Supplementary Figure 3). GFAP co-labeling corroborated astrocytic hypertrophy in HI-NT (Figure 2F, layer II *p* = 0.0024; Figure 2G, layer III *p* = 0.0002 SH-NT vs. HI-NT) with mitigation by HT (Figure 2F, layer II *p* = 0.0301; Figure 2G, layer III *p* = 0.0256, HI-NT vs. HI-HT), showing that cooling stabilizes both GLAST and glial molecular and structural features.

### HT delays epileptogenesis and suppresses seizure burden and spectral power

cEEG revealed a two-phase temporal separation under HT. In HI-NT, seizures emerged ~28 h and peaked ~36 h post-insult; in HI-HT, onset was delayed to ~52 h (Figure 3B), and seizure frequency (Figure 3C, *p* = 0.01005) and duration (Figure 3D, *p* = 0.03973) were reduced. SH-HT piglets displayed brief, cooling-phase discharges peaking ~8 h (Figure 3B, *p* = 0.005)—events likely driven by HT/anesthesia rather than HI injury. PSD analyses showed elevated theta (Figure 3F, 4–8 Hz; *p* = 0.0392), beta (Figure 3G, 12–30 Hz; *p* = 0.0407), and fast gamma (Figure 3B, 70–120 Hz; *p* = 0.0456) in HI-NT, consistent with persistent hyperexcitability. By contrast, HI-HT exhibited profound suppression across these bands (Figures 3F–H, all *p* = 0.0001), a pattern reproduced in supplemental analyses (theta/beta/fast gamma *p* = 0.0016/0.0028/0.0014, Supplementary Figure 6), indicating global network down-regulation. Together, these data link HT, astrocytic GLAST normalization with attenuated cortical excitability and delayed epileptogenesis, perhaps due to restored astroglial glutamate transport.

### Cortical layer resolution coupling between GLAST, neuronal injury, and seizures is restored by HT

Correlation analyses suggested where and how HT re-establishes neuron–astrocyte–network coupling. In HI-NT, ischemic neuron density correlated positively with seizure frequency across layers, maximally in layer III (Figures 4A–C, R^2^ = 0.7799), supporting a link between laminar neuronal loss and epileptogenicity. Under HI-HT, coupling reorganized with weak inverse association in layer I (Figure 4D, R^2^ = 0.5191), no association in layer II (Figure 4E, R^2^ = 0.0031), and a strong positive correlation in layer III (Figure 4F, R^2^ = 0.9561)—suggesting selective preservation and adaptive control in the lamina that most strongly shapes corticocortical synchrony (McGinn and Valiante, 2014; D’Souza and Burkhalter, 2017). While these correlations are robust within our cohort, we acknowledge that the small size limits the generalizability of these associations to a larger population. The high coefficients of determination (R^2^) should be interpreted with caution, as they may reflect the distinct separation between treatment groups rather than a continuous linear relationship in a larger population.

GLAST immunoreactivity metrics tracked with seizure dynamics in a state- and layer-dependent manner. In SH-NT, larger GLAST puncta are associated with lower seizure frequency in layer II (Figure 4G, R^2^ = 0.9868) and shorter duration in layer III (Figure 4H, R^2^ = 0.7677). These changes might reflect homeostatic astrocytic response after HT, as evidently shown-though not statistically significant- by the enlarged GLAST puncta size in cortical layer II/III (Figures 2D,E) and the increased astrocytic coverage in layer II (Figures 2H,J) observed in SH-HT piglets and during overnight anesthesia without HI. In HI-NT, GLAST, and seizure correlations were weak or inconsistent. The lack of association could suggest disrupted glial control. Critically, in HI-HT, GLAST density correlated strongly and positively with both seizure frequency (Figure 4K, R^2^ = 0.9795) and duration (Figure 4L, R^2^ = 0.8746), with progressive strengthening by cortical depth and peaking in layer III (Figures 4M,N). We interpret this as functional reinstatement of astrocytic–neuronal coupling under HT. Perhaps glutamate transporters are correctly positioned and structurally preserved, and their dynamic recruitment scales with residual seizure drive, most detectably in layer III microcircuits (Namiki et al., 2013).

To further interrogate how astrocyte structure relates to laminar neuronal injury, we examined the relationship between GLAST metrics and ischemic neuron density (Figures 4O–T). In HI-NT piglets, meaningful associations were largely restricted to layer II: ischemic neuron density correlated positively with GLAST puncta size (Figure 4O, R^2^ = 0.6427), weakly with coverage (Figure 4P, R^2^ = 0.0963), and inversely with GLAST density (Figure 4Q, R^2^ = 0.3467). This pattern suggests disorganized, possibly maladaptive GLAST hypertrophy in the absence of cooling, with puncta enlargement failing to compensate for diminished GLAST density.

In HI-HT piglets, astrocyte–neuron coupling became more coherent and extended across both layers II and III. In layer II, ischemic burden was strongly and inversely related to GLAST density (Figure 4Q, R^2^ = 0.9104), consistent with preserved GLAST abundance in HI-HT piglets with lower injury severity. In layer III, ischemic neuron density correlated almost perfectly with GLAST puncta size (Figure 4R, R^2^ = 0.9975) and strongly with coverage (Figure 4S, R^2^ = 0.8609). These laminar-specific relationships reinforce the idea that HT restores structured GLAST microdomain organization in proportion to neuronal preservation. Taken together, these analyses suggest that HT not only limits neuronal injury but also re-establishes a coordinated, layer-specific relationship between astrocytic GLAST architecture, neuronal vulnerability, and epileptogenesis. This suggests that GLAST microdomain integrity is a potential regulator of laminar excitability, linking cellular pathology to network-level outcomes in neonatal HIE.

### HI produces complex focal-to-generalized seizure transitions, and HT shifts timing and propagation

High-resolution cEEG segmentation revealed sequential seizure evolution after HI: early low-amplitude bilateral bursts, ramping to high-amplitude discharges with post-ictal depression, and focal onsets (e.g., left posterior somatosensory cortex; Figure 5C, green box) that secondarily generalized over hours to days (e.g., by ~127,000 s on day 5; Figure 5F, green box). Synchronous “leakage spikes” in non-primary channels suggest subthreshold propagation and interareal susceptibility. HT delayed these transitions into the rewarming/recovery window and shortened events, indicating partial disruption of recruitment and spread, consistent with lower PSD and preserved layer III cyto-architecture.

### Focal and generalized seizures have distinct spectral fingerprints, and HT reshapes their expression

Our automated pipeline (majority voting across PSD ratio, entropy, coherence, and Hurst exponent) separated focal (single-channel) from generalized (synchronous multi-channel) spike–wave events. Focal events emphasized delta–theta (1–8 Hz) power (Figures 6K,L); generalized events emphasized beta (12–30 Hz) and slow gamma (30–60 Hz) (Figures 6M,N). Across groups, focal event counts did not differ significantly, though durations clustered around ~5 s with higher inter-animal variability in HI-HT (Figure 6H). Generalized events were more frequent in HI-HT (Figure 6I, *p* = 0.0279) but were shorter and less variable (~3 s) than in other groups (Figure 6J). This finding meshes with the overall seizure burden reduction reported in Figures 3, 4. The generalized spike–wave classifier captures brief, widespread but low-power events under HT (Figures 6F–J), whereas prolonged, high-power ictal episodes that drive burden metrics are suppressed (Figures 3C,D). Moreover, global PSD suppression in HI-HT (Figures 3, 6; Supplementary Figures 6, 8) indicates that these additional generalized detections reflect short-duration, spectrally dampened synchronization rather than more severe seizures. Thus, HT shifts the seizure landscape toward brief, spectrally constrained generalizations while reducing prolonged ictal activity. This increase in generalized event counts does not represent a worsening of epilepsy with HT. These events were significantly shorter and possessed lower spectral power compared to the high-amplitude ictal burdens seen in HI-NT piglets. Furthermore, our automated detection thresholds were applied uniformly across all experimental groups and conditions, ensuring that this shift represents a true change in seizure dynamics rather than detection bias.

### Presynaptic vulnerability persists despite astrocyte and neuronal protection from HT

Synaptophysin staining revealed persistent presynaptic pathology in HI-NT and HI-HT piglets. Puncta density increased across layers I–III (Figures 7D,F,H), but puncta size decreased (Figures 7E,G,I), with a highly selective loss of perisomatic terminals (Figures 7B,C). Because perisomatic inputs are enriched in GABAergic boutons (Szabó et al., 2010; Freund and Katona, 2007; Vereczki et al., 2016; Nagy-Pál et al., 2023), this pattern suggests deficient inhibitory mechanisms even when pyramidal neurons and astrocytes are preserved. HT did not normalize these synaptic vesicle phenotypes, indicating that glutamate transporter rescue and neuronal survival are not sufficient to restore perisomatic inhibition or presynaptic vesicle organization. These data argue for adjunct therapies that stabilize synapses (particularly perisomatic inhibitory networks) alongside HT.

### Piglet–human concordance supports astrocytic GLAST as a translational target

In both human and piglets, we observed superficial astrocytic hypertrophy, perisomatic GLAST clustering, and reduced deeper-layer puncta density, aligning with the laminar pattern of injury and excitability. Our piglet model advanced mechanistic insights into this pathology that were observed in the human cohort. While HT normalized GLAST localization and reduced seizure burden in piglets, the apparent lack of GLAST rescue in HT human infants likely reflects unavoidable heterogeneity in injury severity and duration in the human case study. The piglets are precisely controlled with respect to insult severity and timing. In a clinical setting, the injury onset is often unknown, and the intensity is lacking in clear quantity and is generally believed to be moderate to severe to meet the criteria for cooling. Consequently, the human samples likely reflect a ceiling effect of severe damage that HT could not reverse, rather than a failure of the mechanism itself. Moreover, we were unable to link GLAST pathology directly to seizure history in the human cases due to the absence of continuous EEG data. However, these do not contradict the piglet findings. Despite these human limitations, the shared spatial signature of astrocytic GLAST organization and HT responsiveness across species validates GLAST as a translational hub connecting excitotoxic drive with network hyperexcitability in neonatal HIE.

### Limitations and future directions

While our data demonstrate a strong spatial and temporal association between GLAST preservation and seizure suppression, we explicitly acknowledge that this study establishes correlation rather than direct causality. It remains possible that GLAST remodeling is a downstream consequence of network stabilization provided by HT, rather than the primary driver. Additionally, our correlation analyses yielded high R^2^ values (e.g., >0.95). We are cautious in interpreting these as linear predictors for the general population, given the small sample size (*n* = 4–6). These values likely reflect the robust, binary treatment effect of HT, which creates two distinct clusters of data points (protected vs. unprotected) rather than a continuous spectrum. Although the piglet model reproduces key histopathological and electrophysiological signatures of human HIE, fundamental differences limit direct extrapolation. The timing of injury and intervention in piglets is experimentally controlled and occurs within a defined therapeutic window, whereas human neonatal HIE encompasses variable insult durations, treatment onset, and clinical care heterogeneity that can influence outcome. Moreover, the human cases in this study represent end-stage, fatal pathology likely reflecting irreversible damage, whereas the piglet cohort survives to 7 days, capturing a subacute timepoint where HT is active and adaptive processes are ongoing. The absence of cEEG in the human cases prevents direct correlation of GLAST pathology with seizure history, further constraining translational interpretation. Finally, while high R^2^ values indicate strong clustering by treatment group, they may not reflect continuous relationships in a larger heterogeneous population. These factors must be acknowledged when generalizing from models to clinical scenarios.

To move beyond correlation and establish a causal linkage between astrocytic GLAST function and seizure susceptibility, biochemical studies and gene-targeted interventional studies are required. First, biochemical analyses of functional glutamate transport in regional cortical synaptosomes from NT and HT treated piglets need to be done for correlations with seizure patterns. Then, astrocyte-specific manipulations using viral vectors, such as recombinant AAVs with promoters (GFAP) driving GLAST to enforce expression or virus driving siRNA to knock down GLAST expression, during HI and HT, to adjust the neurodegeneration and seizure outcomes in HI piglets with and without HT. Other *in vivo* experiments could focus on longitudinal imaging of extracellular glutamate (e.g., with genetically encoded sensors such as iGluSnFR) to directly quantify glutamate clearance dynamics in relation to seizure onset. Combinatorial therapeutic strategies pairing HT with small molecule drugs that enhance glutamate transporter expression or function (e.g., *β*-lactam antibiotics shown to upregulate EAATs) warrant investigation for additive neuroprotection and seizure suppression. Additionally, exploring presynaptic stabilizers that reinforce inhibitory networks could address persistent synaptic vesicle deficits not rescued by HT (Pajarillo et al., 2019). These avenues will clarify the mechanistic hierarchy and therapeutic potential in neonatal HIE.

## Conclusion

This study identifies astrocytic GLAST remodeling as a morphological change associated with HI injury and seizure susceptibility in neonatal HIE and demonstrates that HT preserves GLAST remodeling and reduces seizure burden in a translational piglet model. GLAST aggregation and reduced puncta density seen in human HIE were faithfully reproduced after HI in piglets and normalized by HT. This astrocytic preservation paralleled delayed seizure onset, reduced seizure burden, and layer-specific neuronal protection, particularly in layer III of the somatosensory cortex, where GLAST density correlated with both seizure frequency and duration. Spectral EEG features distinguished focal and generalized seizures, and HT shifted cortical seizure dynamics toward brief, low-power, spectrally suppressed episodes. Despite this, presynaptic vesicle disorganization persisted, indicating that synaptic restoration remains an unmet therapeutic target. The translational concordance of astrocytic GLAST remodeling between human HIE and the piglet model underscores the potential of GLAST as being relevant to the evolution of neonatal brain injury and seizures. In clinical practice, encouraging proton magnetic resonance spectroscopy (1H-MRS) usage can increase detection of specific areas of the brain with elevated glutamate–glutamine levels in neonates with HIE that correlate positively with seizure severity. Early intervention of GLAST dysregulation via brain scanning technology could stratify infants at high risk for refractory seizures despite therapeutic hypothermia. Our findings suggest that augmenting HT with strategies that promote astrocytic glutamate clearance and reinforce inhibitory synaptic networks may enhance seizure control and neuroprotection. These insights encourage integration of astrocyte-directed approaches into evolving HIE treatment paradigms and highlight the importance of preserving glial function alongside conventional neuronal targets.

## Data Availability

The data that support the findings of this study are available from the corresponding author upon reasonable request.
