# Supplementary material for: Prominent astrocytic GLAST pathology occurs in newborn human and piglet hypoxic–ischemic encephalopathy: modeling relationships among laminar neuropathology, seizures, and therapeutic hypothermia
Source: Front Cell Neurosci. 2026 Feb 24;20:1758411. doi: 10.3389/fncel.2026.1758411 (PMC12971461; doi:10.3389/fncel.2026.1758411)
Supplement: Supplementary file 1 [file Table_1.DOCX]

**Supplemental figures and legends**

**Supplemental figure 1. GLAST and GFAP Co-localization in human a cortical astrocytes (supplemental to figure 1).**

In the cortical layers, protoplasmic astrocytes are identified by GFAP (red) highlighting their cell bodies and processes. GLAST (green) is localized to the membranous surfaces of GFAP-positive astrocytes, confirming astrocytic expression of GLAST in the human cortex.

**
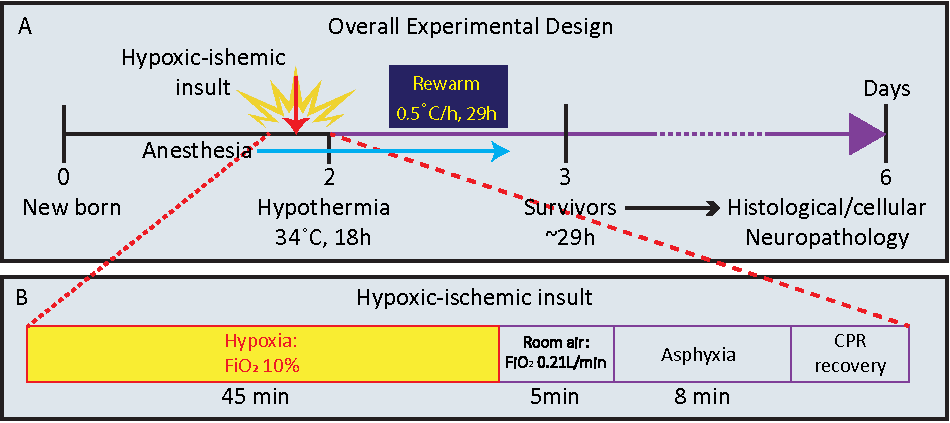
**

**Supplemental figure 2. Diagram summarizing the 2-3 day old piglet model with cEEG recording, hypoxic-ischemic insult and hypothermia treatment (Supplemental to Figure 2).**

A) The overall experimental design involved cEEG recordings in neonatal piglets subjected to hypoxic-ischemic insult, followed by resuscitation and therapeutic hypothermia for 29 hours and a survival lasting for another five days. The piglets remained under anesthesia for the entire duration of the sham and HI procedures and the 29 hours of hypothermia or normothermia. Afterwards, they were awake until euthanasia. All piglets underwent transcardial perfusion for subsequent neuropathological analysis. B) The schematic diagram illustrates the hypoxic-ischemic insult protocol and the subsequent resuscitation procedures.

**
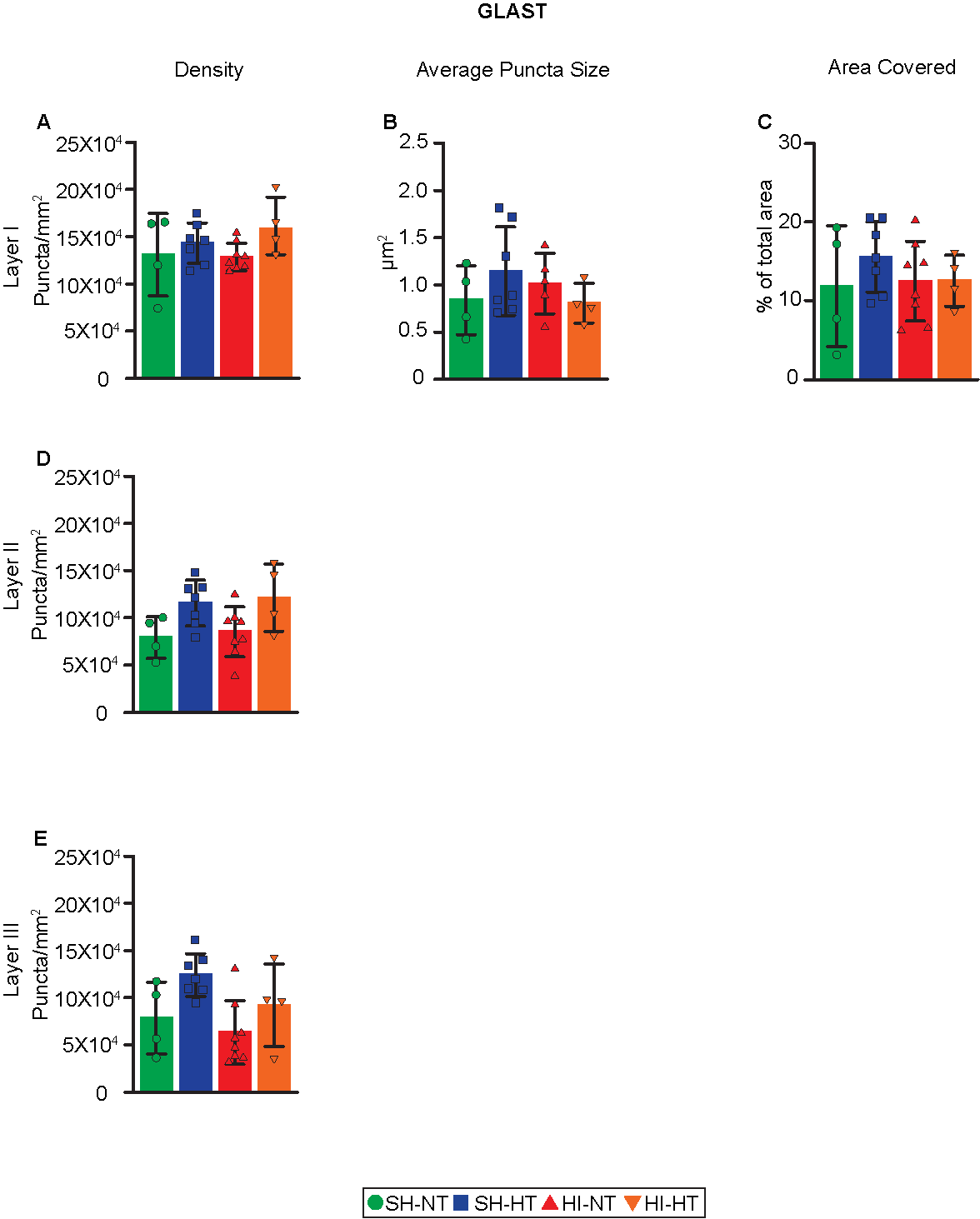
**

**Supplemental figure 3. Layer-specific GLAST localization and correlation with seizure metrics in the somatosensory cortex of piglets (supplemental to figure 2).**

A-C) Quantification of GLAST positive puncta density, particle sizes, percent of cross-sectional somal area covered by GLAST in layer I of somatosensory cortex. No significant change was observed among all groups of piglets. D) Quantification of GLAST density in layer II of somatosensory cortex. No significant change was observed. E) Quantification of GLAST density in layer III of somatosensory cortex. No significant change was observed. Statistical analysis: Normality was assessed using the Shapiro-Wilk test. Normal-distributed samples were analyzed using an ANOVA with a multiple comparison test or an unpaired two-tailed Welch’s test. Lognormal-distributed samples were analyzed using the Kruskal-Wallis test followed by a Dunn’s multiple comparison test or a Mann-Whitney test with 95% confidence. **p*<0.05. *p* values indicated.

**
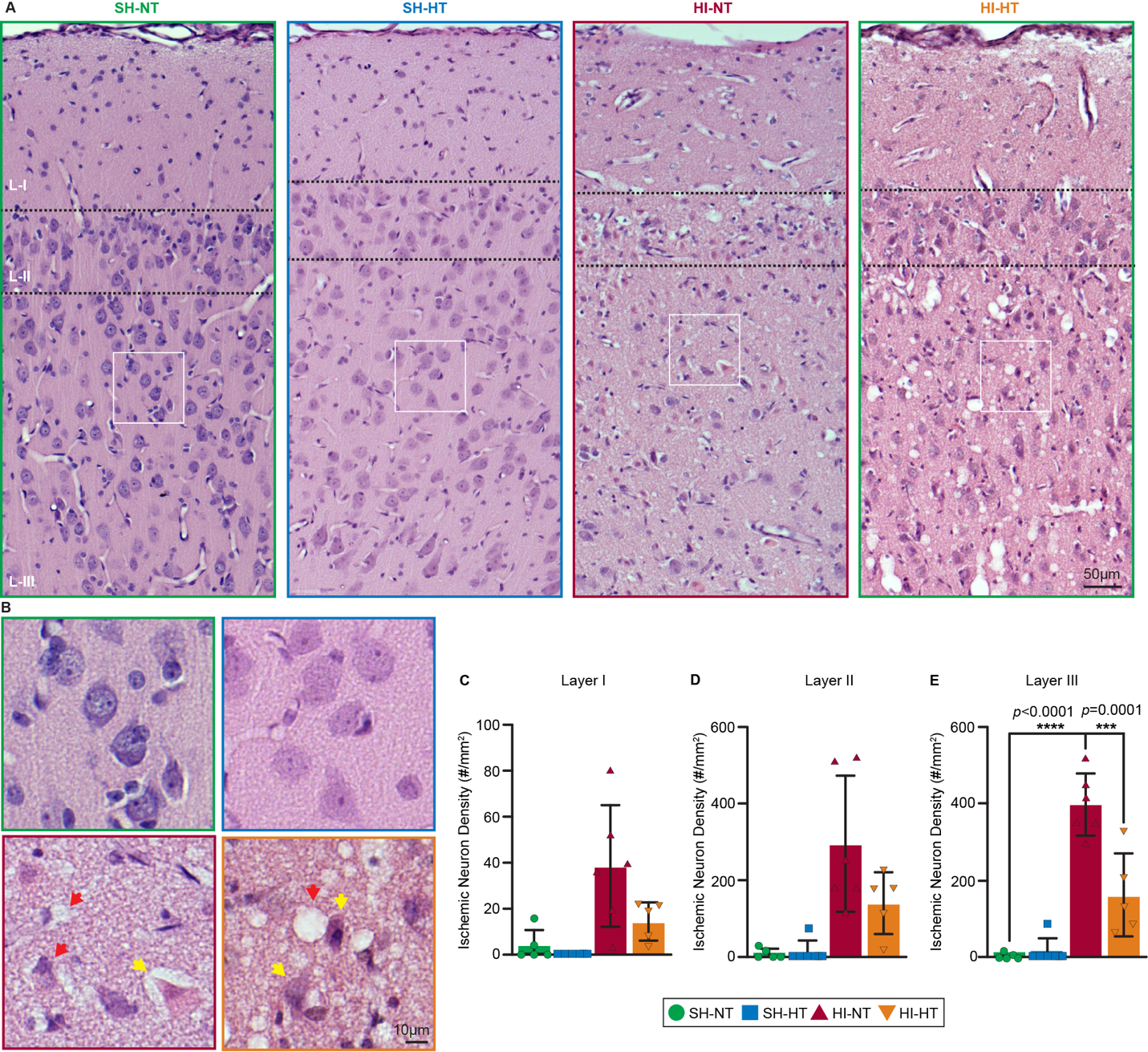
**

**Supplemental figure** 4. Neocortical Neuropathology in HI Piglets is Suppressed by HT

A) Representative low-magnification images of H&E staining showing layer I through layer III of somatosensory cortex in each piglet treatment group. B) Representative high-magnification images of H&E staining showing the boxed areas of in A containing representative ischemic-necrotic neurons in layer III. C-E) Graphs of ischemic neuron densities (mean ± SD) in each layers I, II, and III of somatosensory cortex. Piglet treatment groups are color coded. HT treatment reduced significantly ischemic neuronal density only in layer III. Statistical analysis: Normality was assessed using the Shapiro-Wilk test. Normal-distributed samples were analyzed using an ANOVA with a multiple comparison test or an unpaired two-tailed Welch’s test. Lognormal-distributed samples were analyzed using the Kruskal-Wallis test followed by a Dunn’s multiple comparison test or a Mann-Whitney test with 95% confidence. **p*<0.05, ***p*<0.001, ****p=*0.0001. *p* values indicated.

**
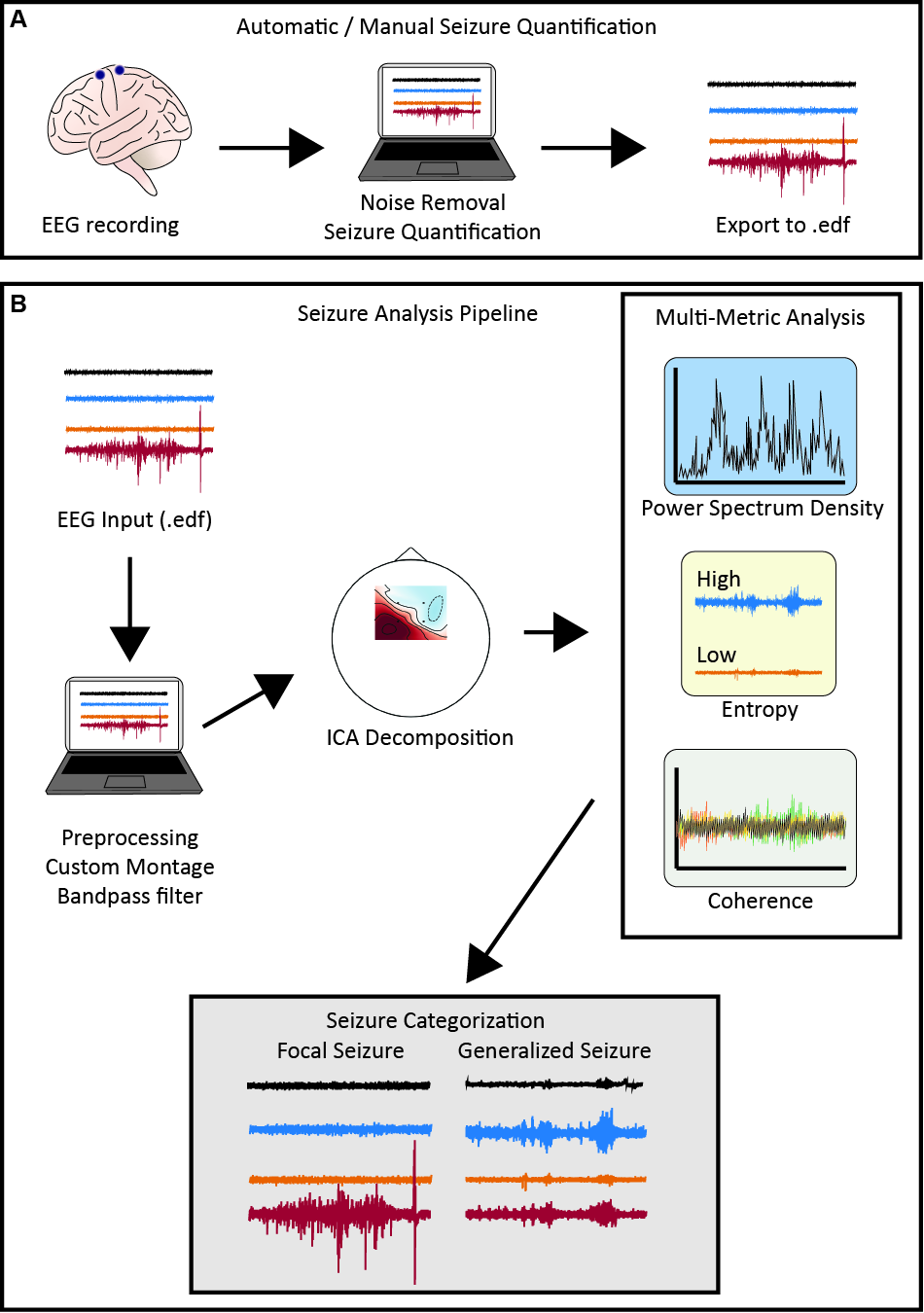
**

**Supplemental figure 5. Flowchart summarizing the seizure analysis and categorization pipeline (Supplemental to Figure 3 and Figure 6).**

A) Signal processing with noise reduction and automatic seizure quantification with Notocord-HEM® and signal export to .edf for analysis. B) EEG signals were preprocessed and segmented into epochs. Spike-wave form was used to detect seizure by program automation. Detected events were classified as generalized or focal seizures using five independent metrics (PSD ratio, entropy, RMS, coherence, Hurst exponent), followed by majority voting. ICA was applied to each data condition (seizures and baseline) to visualize independent components contributing to seizure dynamics. Final metrics and classifications were exported as a comprehensive Excel report.


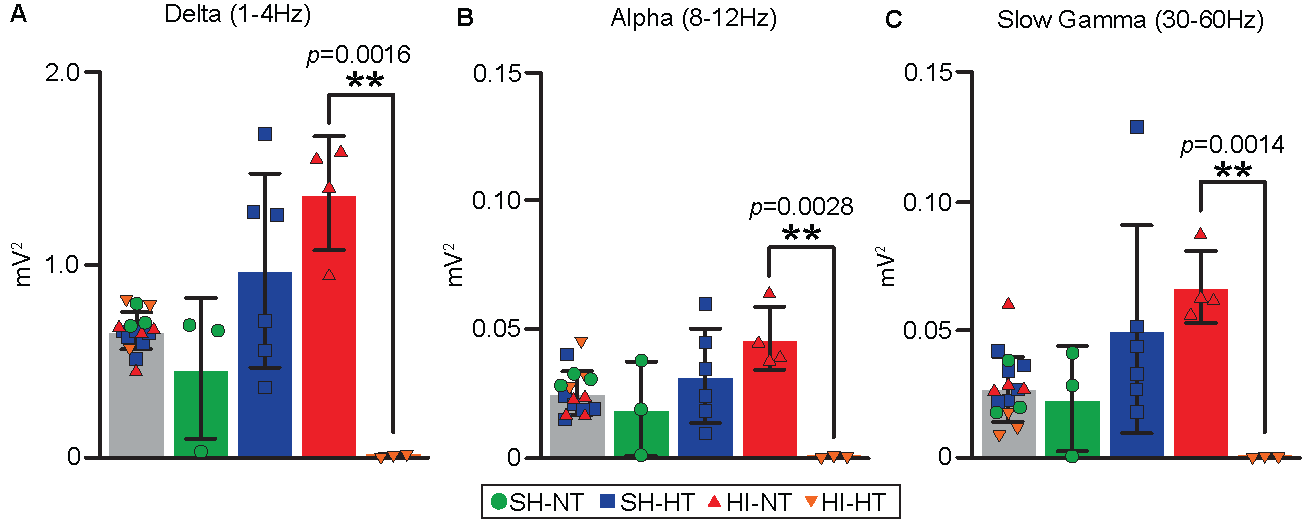


**Supplemental figure 6. Power spectral density (PSD) analysis of ictal seizure evets (Supplemental to figure 3).**

A-C) Normalized (PSD compared against baseline) delta wave (A, 1-4 Hz), alpha wave (8-12 Hz), and slow gamma wave (30-60 Hz). Statistical analysis was done with a Shapiro-Wilk normality test, followed by Tukey’s multiple comparisons test or a Kruskal-Wallis test followed by Dunn’s multiple comparisons test with 95% confidence. **p*<0.05. *p* values indicated.


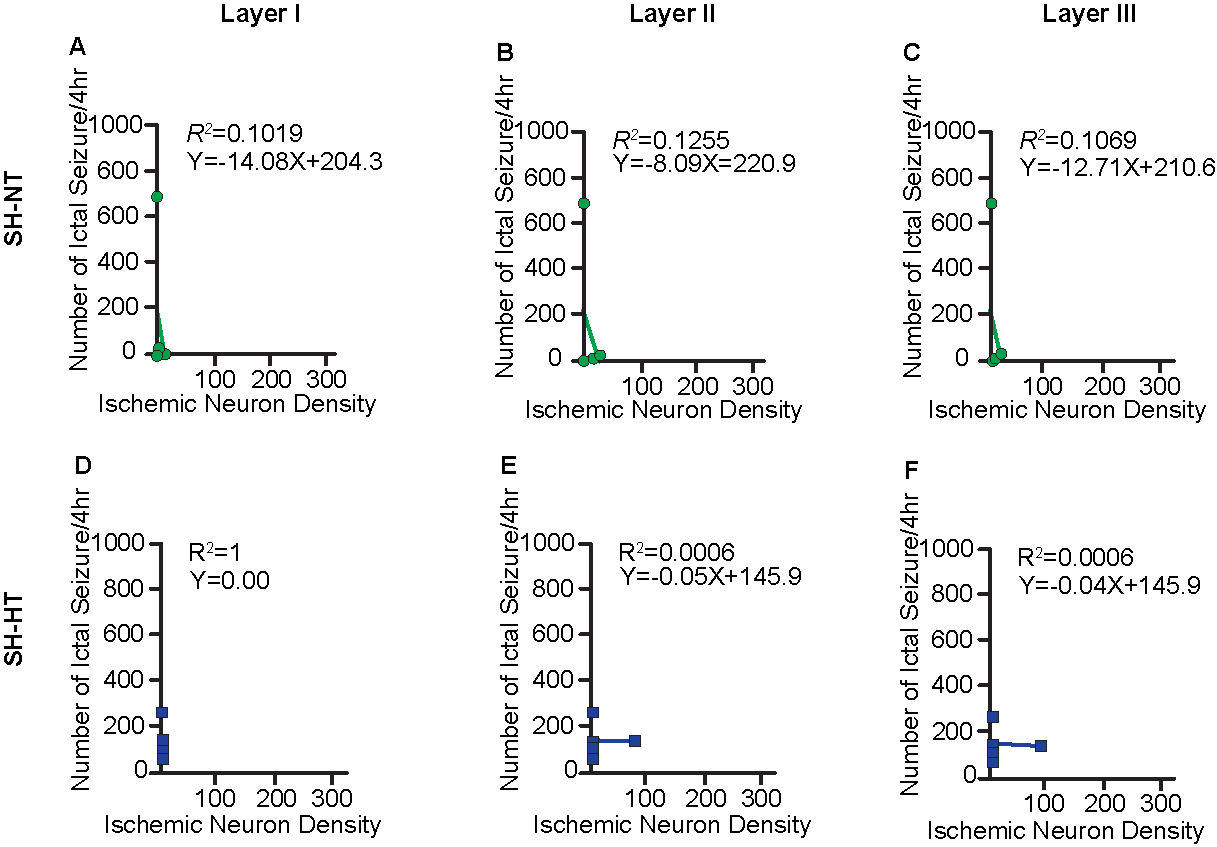


**Supplemental figure 7. Layer-specific ischemic-necrotic neuron density in somatosensory cortex correlates with average spontaneous recurrent seizure (SRS) frequency during 32-44 hr after the HI insult. (Supplemental to figure 4).**

A-C) Cortical layer specific neuropathology (ischemic neuron density) correlations with ictal seizure frequency in SH-NT piglets. SH-NT piglets had negative correlation with steep slope in all layers with low R^2^ values. D-F) Cortical layer specific neuropathology (ischemic neuron density) correlations with ictal seizure frequency in SH-HT piglets. SH-HT piglets had a slight negative correlation in all layers with very low correlation and negligible slope in all layers.


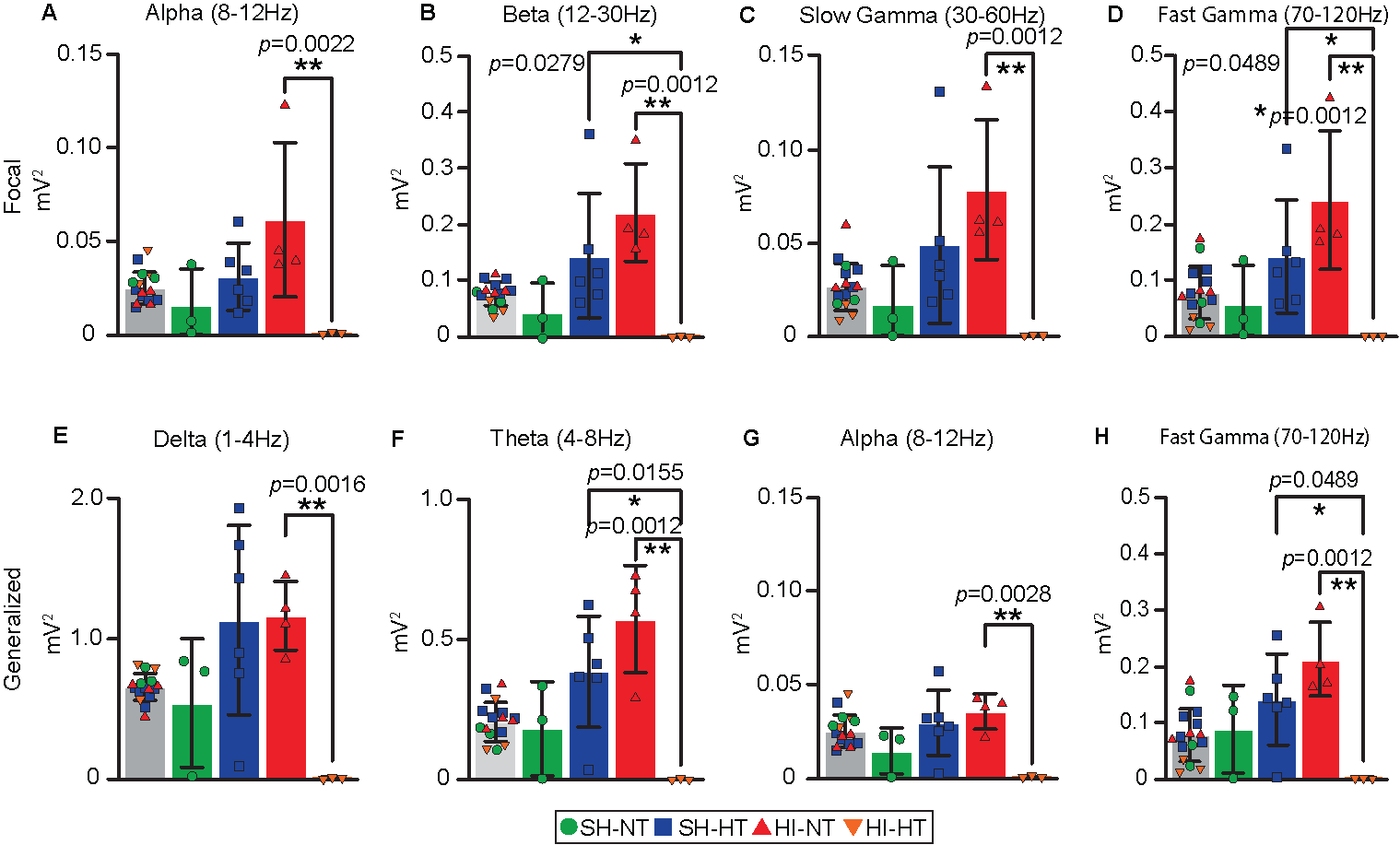


**Supplemental figure 8. PSD Focal/General Spike-Wave PSD (Supplemental to figure 6).**

A-H) PSD analysis of focal or generalized spike-wave events. Piglet treatment groups are color coded. Gray bar represents invariant average baseline of all treatment groups. A-D) Normalized PSD from focal spike-wave events. A) alpha (8-12 Hz) band, B) beta (12-30 Hz) band, C) slow gamma (30-60 Hz) band, D) fast gamma (70-120 Hz) band, E-H) Normalized PSD from generalized spike-wave events. E) delta (1-4 Hz), F) theta (4-8 Hz), G) alpha (8-12 Hz) band, H) fast gamma (70-120 Hz) band. Statistical analysis was done with a Shapiro-Wilk normality test, followed by Tukey’s multiple comparisons test or a Kruskal-Wallis test followed by Dunn’s multiple comparisons test with 95% confidence. Significance set at *p*<0.05 with *p* values indicated.


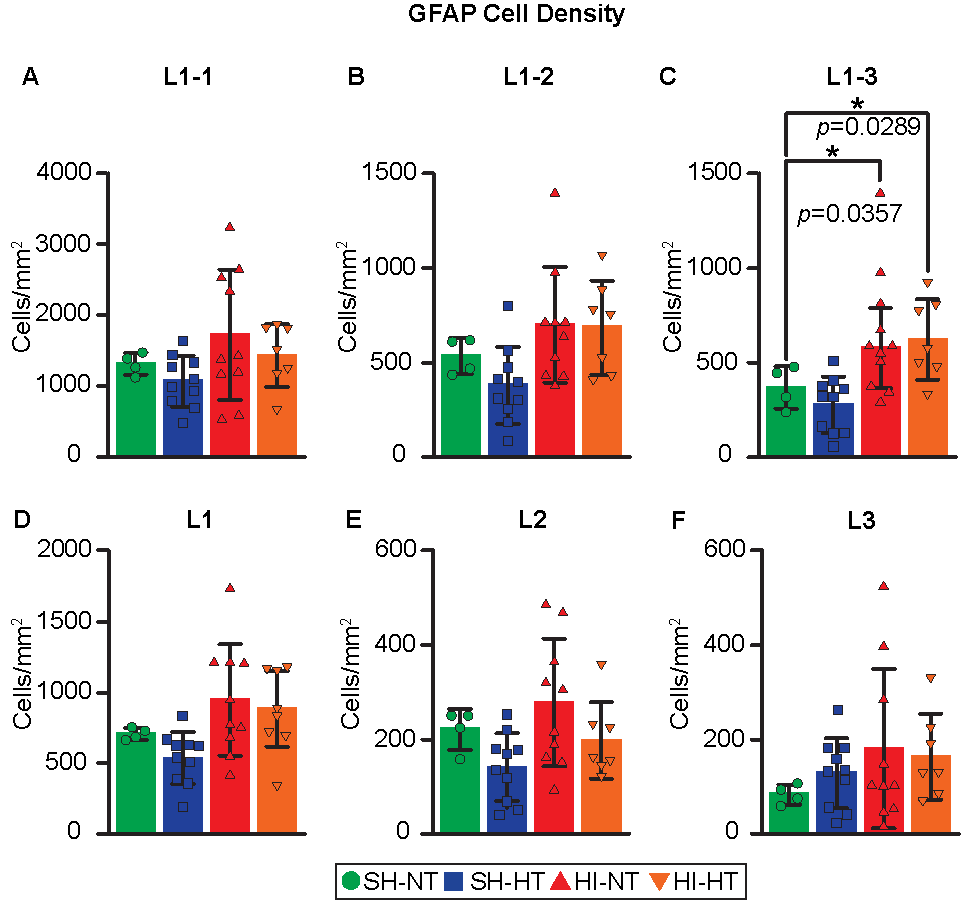


**Supplemental figure 9. Layer-specific GFAP positive astrocyte cell density in somatosensory cortex (supplemental to figure 2).**

A-C) layer I of somatosensory cortex was divided to 3 separate equidistance layers. L1-1 being superficial and L1-3 is superior to layer II. Astrocyte density was increased through all layers in HI-NT piglets. A particularly significant increase was observed in L1-3 of layer I. D-F) Total GFAP positive astrocyte cell density in layer I (D), layer II (E), layer III(F). Astrocyte density was generally increased through all layers in HI-NT piglets, but it was not statistically significant. Statistical analysis was done by Welch’s T test to compare each group against SH-NT group. **p<0.001, *p<0.0, p values are indicated.
